# Interpretable machine learning models for identifying cognitive impairment in middle-aged and older adults with mild and severe insomnia: development, temporal validation, and clinical external validation

**DOI:** 10.3389/fnagi.2026.1855864

**Published:** 2026-06-23

**Authors:** Qilong Wang, Siheng Ma, Jianpeng Zhao, Xin Qi, Dongmei Ma, Sha Liu, Lei Zhang, Chen Wang, Yan He, Dongrong Zhao

**Affiliations:** 1 Department of Psychiatry, Gansu Provincial People’s Hospital, The First Clinical College of Gansu University of Chinese Medicine, Lanzhou, Gansu, China; 2 Department of Psychiatry, Xijing Hospital, The Fourth Military Medical University, Xi’an, Shaanxi, China

**Keywords:** cognitive impairment, insomnia, LightGBM, machine learning, SHAP

## Abstract

**Background:**

Cognitive impairment is an important health issue in middle-aged and older adults, and insomnia may be associated with increased cognitive vulnerability. However, models specifically designed to identify cognitive impairment in individuals with different severities of sleep-duration-defined insomnia remain limited. This study aimed to develop and validate interpretable machine learning models for current cognitive impairment identification in mild and severe insomnia subgroups.

**Methods:**

Data from CHARLS 2015 were used as the development cohort, CHARLS 2011 as the cross-wave temporal validation cohort, and clinical data from Gansu Provincial People’s Hospital as the clinical external validation cohort. Participants with insomnia were stratified into mild and severe subgroups according to self-reported nighttime sleep duration. LASSO regression was used for feature selection, and candidate machine learning algorithms were compared for model selection. The selected LightGBM model was further evaluated using Bayesian optimization and optimized-threshold analysis. Model performance was assessed using AUROC, Brier score, calibration curves, decision curve analysis, and SHAP-based interpretability analysis.

**Results:**

The development, cross-wave temporal validation, and clinical external validation cohorts included 5,500, 4,231, and 500 participants, respectively. LightGBM showed the most balanced overall performance. In the mild insomnia subgroup, LightGBM achieved AUROCs of 0.772, 0.749, and 0.748 across the three cohorts; in the severe insomnia subgroup, the corresponding AUROCs were 0.763, 0.757, and 0.750. Bayesian optimization produced comparable external validation discrimination, while optimized-threshold analysis improved threshold-dependent classification performance. SHAP analysis suggested different feature contribution patterns across insomnia severity.

**Conclusion:**

LightGBM provided moderate and relatively stable performance for identifying current cognitive impairment risk in both insomnia subgroups. Combined with SHAP interpretation and online calculators, these models may support auxiliary screening, preliminary risk stratification, and referral prioritization for formal cognitive assessment, but should not be interpreted as standalone diagnostic tools or tools for predicting future incident cognitive impairment in sleep medicine settings.

## Graphical abstract

**Figure:**
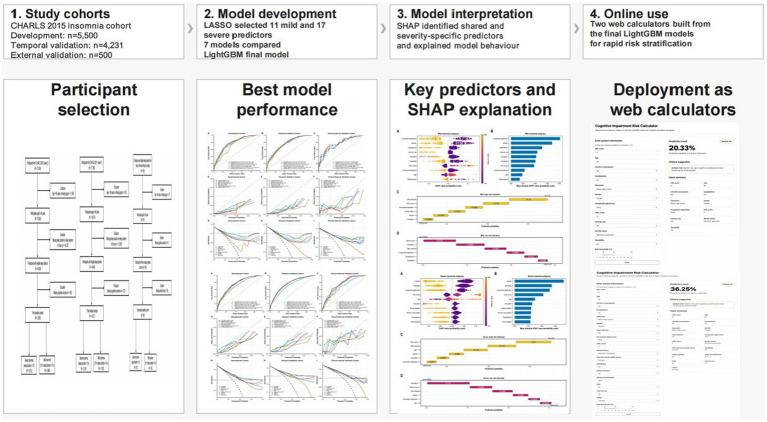

## 1 Introduction

Cognitive impairment is a common and significant health issue among the elderly, and it represents a critical clinical stage in the onset and progression of dementia. As the global population ages at an accelerating rate, the burden of cognitive impairment and related diseases continues to rise. The GBD 2019 Dementia Collaboration Group predicts that the global number of people with dementia will increase from 57.4 million in 2019 to 152.8 million by 2050 (GBD 2019 Dementia Forecasting Collaborators, 2019). In China, a nationwide cross-sectional study revealed that approximately 15.07 million people aged 60 and older have dementia, with an additional 38.77 million suffering from mild cognitive impairment (Jia et al., 2020). Cognitive decline not only impairs memory, executive function, and activities of daily living but also increases the burden of care, the consumption of healthcare resources, and the risk of subsequent progression to dementia (Marshall et al., 2011; Paradise et al., 2015; Gläser et al., 2025). Therefore, the preliminary identification of individuals at high risk for cognitive impairment and the implementation of stratified management have become critical priorities in geriatrics, neuropsychiatry, and public health.

Insomnia is a common sleep disorder among older adults and an important potential factor affecting cognitive health. A meta-analysis showed that the prevalence of insomnia is approximately 15.0% (Cao et al., 2017). As people age, middle-aged and older adults are more prone to shorter sleep duration, difficulty maintaining sleep, and impaired daytime functioning; these changes may further affect attention, memory, executive function, and overall cognitive performance (Li et al., 2018). Previous studies have shown that insomnia is closely associated with cognitive aging; individuals with insomnia have an increased risk of cognitive decline or Alzheimer’s disease and may exhibit impairments across multiple cognitive domains (Zhang et al., 2024). Further research has found that the severity of insomnia symptoms is associated with poorer overall cognitive and memory performance, suggesting that the severity of insomnia may influence cognitive risk (Baril et al., 2022). Therefore, identifying the risk of cognitive impairment in individuals with insomnia, while also considering risk heterogeneity across different severity levels of insomnia, is of great significance for early screening and stratified intervention in the context of sleep medicine.

Machine learning methods have been applied to the early screening and risk identification of cognitive impairment and mild cognitive impairment (Li et al., 2023; Wu et al., 2025). Although recent studies have used multi-wave CHARLS data and other national cohorts for longitudinal prediction or cognitive trajectory modeling in older adults (Cao et al., 2026; Long et al., 2026), their focus differs from the present study, which aimed to identify current cognitive impairment within a sleep-duration-defined insomnia population. However, existing studies have primarily focused on the general elderly population, and sleep-related information has typically been incorporated into models only as candidate predictors or as a general indicator of sleep disorders. Studies that build separate models for populations with different severities of insomnia and compare the heterogeneity of cognitive impairment-related predictor patterns remain limited. Therefore, this study developed an interpretable machine learning framework to construct separate cognitive impairment identification models for individuals with mild and severe insomnia and evaluated their generalization performance using a cross-wave temporal validation cohort and a clinical external validation cohort. Furthermore, by integrating the SHAP method and an online risk calculator, this study provides an auxiliary tool for preliminary identification of current cognitive impairment risk, prioritization for formal cognitive assessment, and stratified management in sleep medicine settings.

## 2 Method

This study was reported in accordance with the TRIPOD-AI guidelines, namely the Transparent Reporting of a Multivariable Prediction Model for Individual Prognosis or Diagnosis + Artificial Intelligence, for the development, internal validation, and external validation of machine learning-based identification models (Collins et al., 2024).

### 2.1 Participants

**Figure 1:**
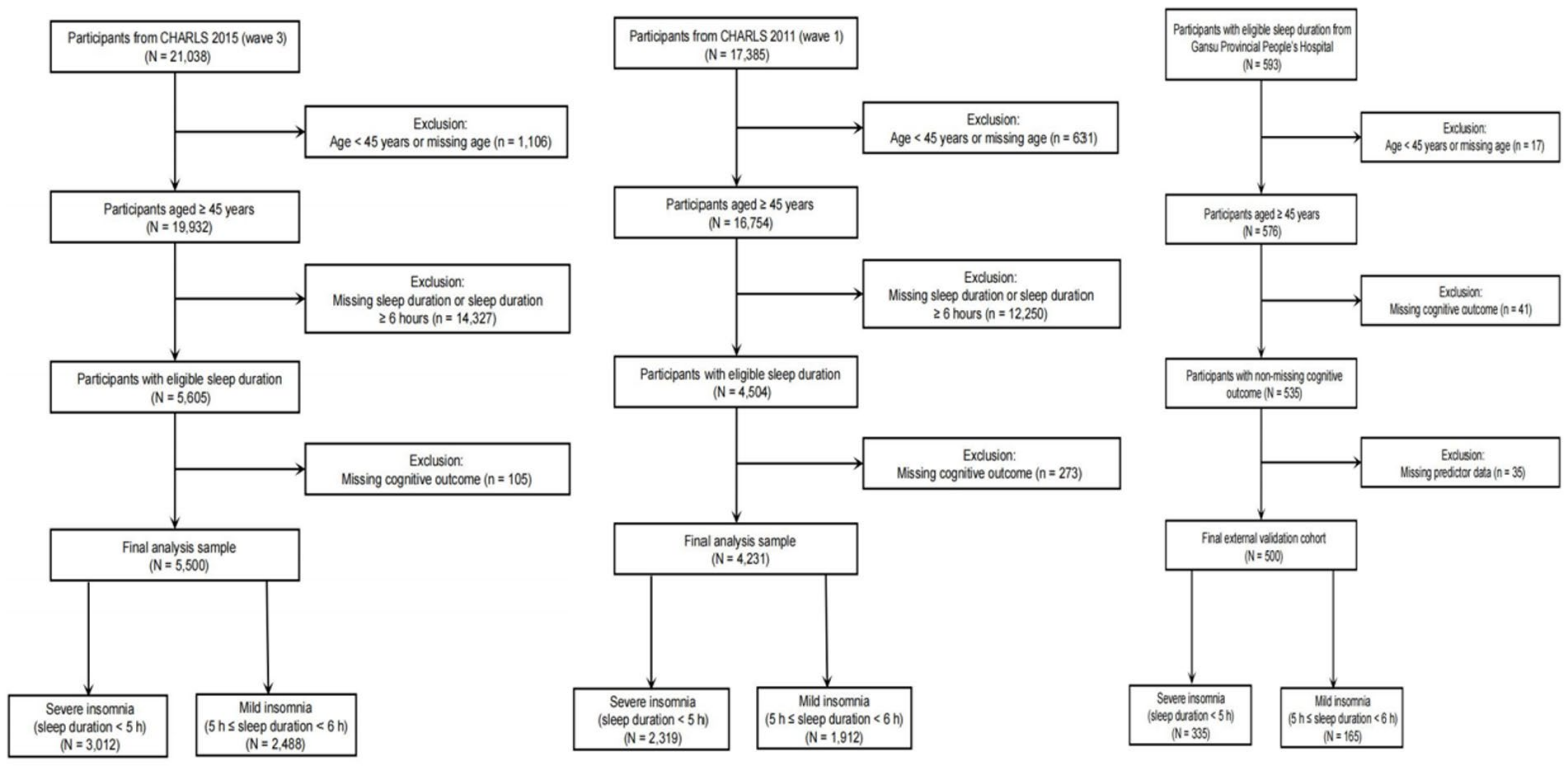
Flowchart of participant selection for the development, temporal validation, and clinical external validation cohorts.

The data for this study were drawn from the third wave of the 2015 China Health and Retirement Longitudinal Study (CHARLS). CHARLS is a nationally representative longitudinal study of Chinese adults aged 45 and older, which collects a wealth of demographic, health, and socioeconomic information (Zhao et al., 2014). The CHARLS study was approved by the Ethics Committee of Peking University (Approval No.: IRB00001052-11015), and all participants signed written informed consent forms. To evaluate the model’s robustness and generalizability, this study included two validation cohorts that were not used for model development. The first cohort was derived from the 2011 CHARLS baseline survey and served as a cross-wave temporal validation cohort to assess the model’s transportability across different CHARLS survey waves, rather than to evaluate prospective prediction of future cognitive impairment; the second cohort was derived from Gansu Provincial People’s Hospital and served as a clinical external validation cohort to assess the model’s external validity across different research settings and populations. The hospital cohort was a cross-sectional study; participants were consecutively enrolled between July 1, 2025, and March 1, 2026. The study was approved by the Ethics Committee of Gansu Provincial People’s Hospital (Approval No. 2025–472), and all participants provided written informed consent before enrollment. Based on the predefined inclusion and exclusion criteria, a total of 5,500 participants were ultimately included in the development cohort, 4,231 in the temporal validation cohort, and 500 in the clinical external validation cohort. The participant screening process is shown in Figure 1. Data on candidate predictors were collected via a structured questionnaire, including demographic characteristics, health status, functional status, and lifestyle factors. The severity of insomnia was operationally classified based on the self-reported average actual nighttime sleep duration. In CHARLS, this variable is primarily derived from the question: “On average, how many hours of sleep did you actually get each night over the past month?” Based on previous studies and sleep duration classification criteria (Vgontzas et al., 2013; Johnson et al., 2021; Gu et al., 2010; Wang et al., 2026), individuals with nightly sleep duration of less than 6 h were classified as having sleep-duration-defined insomnia for epidemiological subgrouping, and were further stratified as follows: sleep duration of ≥5 h but <6 h was defined as mild insomnia, and sleep duration of <5 h was defined as severe insomnia. This classification should be interpreted as a sleep-duration-based operational definition of insomnia severity rather than a formal clinical diagnosis of insomnia disorder. Because CHARLS did not include detailed clinical sleep assessments, such as sleep latency, nocturnal awakenings, early morning awakening, duration of insomnia symptoms, daytime functional impairment, standardized sleep quality scales, or objective sleep monitoring, this study could not capture the full clinical complexity of insomnia. This variable was used solely for participant screening and subgroup classification and was not included in the model as a predictor variable. The present models were developed for current cognitive impairment identification within the predefined sleep-duration-defined insomnia population and were not intended to estimate cognitive impairment risk relative to normal sleepers. The sample size for this study was determined by the total number of available cases from each data source that met the predefined inclusion and exclusion criteria. The sample sizes and the number of outcome events in the development cohort, temporal validation cohort, and clinical external validation cohort were sufficient to meet the requirements for model development, validation, and performance evaluation.

### 2.2 Measurement of cognitive impairment/outcome

Cognitive function in CHARLS is assessed using two dimensions: episodic memory and executive function. Episodic memory is measured using immediate and delayed recall tests involving 10 Chinese words; the score is the average of the two, ranging from 0 to 10 points (Wei et al., 2018; Lei et al., 2012). Executive function is assessed using the Telephone Interview for Cognitive Status (TICS) and a figure-drawing test; the score ranges from 0 to 11 points (Pan et al., 2022). The overall cognitive function score is the sum of the scores for episodic memory and executive function, ranging from 0 to 21 points; a higher score indicates better cognitive function (Li et al., 2022). Based on previous studies (Jak et al., 2009), an overall cognitive function score <6 was defined as cognitive impairment, and ≥6 as normal cognition, serving as the binary outcome variable in this study. The external validation cohort used the same cognitive assessment methods and cutoff values as in CHARLS to ensure comparability of outcome definitions across cohorts.

### 2.3 Candidate predictors

The candidate predictor variables included in this study encompassed sociodemographic characteristics, behavioral and lifestyle factors, and health-related indicators. Sociodemographic characteristics included age, gender, educational attainment, household registration status, marital status, employment status, living arrangements with children, and household size. Behavioral and lifestyle factors included smoking status, alcohol consumption, internet use, and social engagement. Health-related indicators include hypertension, dyslipidemia, diabetes, cancer, chronic lung disease, liver disease, heart disease, stroke, kidney disease, digestive system diseases, arthritis, asthma, history of falls, history of hip fractures, and the use of antidiabetic and antihypertensive medications. In addition, physical and functional indicators include body mass index (BMI), waist circumference, number of chronic conditions, activities of daily living (ADL) scores, instrumental activities of daily living (IADL) scores, life satisfaction, self-rated health status, hearing, and vision. ADLs consist of six activities: dressing, bathing, eating, getting out of bed, using the toilet, and controlling bowel and bladder functions; IADLs consist of five activities: housekeeping, cooking, shopping, making phone calls, and managing finances (Silverstein et al., 2020). Each item is scored based on the presence or absence of difficulty; the higher the total score, the more severe the functional impairment. ADL and IADL scores were included as routinely available functional status indicators; however, given their potential clinical overlap with cognitive impairment, they were interpreted as concurrent functional correlates for risk stratification rather than as causal or temporally antecedent predictors. The number of chronic conditions is defined as the total number of chronic diseases diagnosed by a physician and self-reported by the participant. Life satisfaction, self-rated health status, hearing, and vision are all assessed based on information self-reported by the participants. Detailed definitions and coding of the study variables are provided in Supplementary Table 1.

### 2.4 Data preprocessing and feature selection

For missing predictor variables in the development cohort, this study employed multiple imputation by chained equations (MICE) and applied this method only within the training data to prevent information leakage. Continuous and categorical variables were interpolated using appropriate methods, respectively, while outcome variables were not included in the interpolation. Prediction results for the temporal validation cohort were obtained by averaging across multiple interpolation models; the external clinical validation cohort included only complete cases. Continuous and categorical variables were incorporated into the modeling process according to their data types and were encoded and standardized as required by the models.

To address the issues of multicollinearity and overfitting in high-dimensional feature spaces, this study employed the Least Absolute Shrinkage and Selection Operator (LASSO) regression for dimensionality reduction and feature selection. LASSO analysis was performed separately for the mild and severe insomnia subgroups based on their respective candidate predictors, and the optimal penalty parameter *λ* was determined using 10-fold cross-validation. By applying a penalty term to the regression coefficients, LASSO shrinks some coefficients to zero, thereby selecting the variables most strongly associated with the outcome. The cross-validation curves and coefficient path plots are shown in Supplementary Figures 1, 2, respectively. To improve model interpretability, reduce feature dimensionality, and enhance comparability among different algorithms, this study first applied LASSO regression to screen features in the two insomnia subgroups, and then constructed and compared different machine learning models based on the screened feature sets. Ultimately, 11 features were retained for the mild insomnia group: IADL score, ADL score, gender, alcohol consumption, internet use, age, marital status, household registration type, educational level, social participation, and dyslipidemia. For the severe insomnia group, 17 features were retained: IADL score, ADL score, gender, alcohol consumption, age, heart disease, self-rated health status, internet use, dyslipidemia, marital status, household registration type, kidney disease, waist circumference, educational level, history of falls, vision, and social participation. These features were subsequently used for model development and performance evaluation. The variance inflation factor values of the predictors retained for the mild and severe insomnia subgroups are presented in Supplementary Tables 2, 3, respectively. Because ADL and IADL scores may partly reflect functional consequences associated with cognitive impairment, their inclusion should be understood as part of a pragmatic cross-sectional screening framework. The selected features were not interpreted as causal determinants or temporally preceding risk factors. The results of the correlation analysis for the final predictor variables are shown in Supplementary Figure 3.

### 2.5 Model development and evaluation

After feature selection, this study constructed cognitive impairment identification models separately for the mild and severe insomnia subgroups. The present models were developed for cross-sectional risk identification and auxiliary screening of current cognitive impairment rather than for predicting incident cognitive impairment at a future time point. The machine learning algorithms included in the comparison were logistic regression (LR), support vector machines (SVM), extreme gradient boosting (XGBoost), light gradient boosting machine (LightGBM), random forest (RF), extra trees, and GLMNet. These algorithms were used as candidate models for initial model comparison and final model selection. Logistic regression (LR), as a traditional statistical learning method, served as the baseline model for comparison with other machine learning algorithms. The training, hyperparameter tuning, and performance evaluation of all models were conducted within the development pipeline using 5-fold stratified cross-validation; the validation and external validation pipelines were used solely to assess the models’ generalization capabilities on independent data. Models requiring hyperparameter tuning were optimized using 5-fold cross-validation within the development cohort, with the area under the receiver operating characteristic curve (AUROC) serving as the primary tuning metric. Given the uneven distribution of outcome categories, class-imbalance handling was incorporated into the model development workflow. Where applicable, the Synthetic Minority Over-sampling Technique (SMOTE) was used to enhance the model’s ability to identify minority-class cases. To avoid information leakage, SMOTE was not applied to the entire development cohort before cross-validation. Instead, it was applied only within the training folds during each round of 5-fold stratified cross-validation. In each fold, the held-out validation fold was kept in its original class distribution and was not used during oversampling. SMOTE was not applied to the held-out validation folds, temporal validation cohort, or clinical external validation cohort; therefore, all performance metrics were calculated on data that did not contain synthetic samples. All models output the model-estimated probability of being classified as having current cognitive impairment based on variables measured within the same survey wave. For the temporal validation cohort and the external validation cohort, prediction probabilities were generated separately for each imputed dataset, and the average of the probabilities from each imputation was taken as the participant’s final prediction result. Model performance was evaluated based on three aspects: discriminatory power, calibration, and clinical utility. Discriminatory power was assessed using ROC curves, AUROC, accuracy, precision, recall/sensitivity, specificity, and F1 score; calibration was assessed using calibration curves, Brier score, calibration intercept, and calibration slope; clinical utility was assessed using decision curve analysis (DCA) to evaluate the net benefit at different threshold probabilities. The ROC curve, AUROC, Brier score, calibration metrics, and DCA were all calculated based on continuous predictive probabilities; accuracy, precision, recall, specificity, and the F1 score were calculated based on a predefined classification threshold of 0.5 to ensure consistency in comparisons across different models. Based on a comprehensive comparison of these metrics, the optimal models for the mild and severe insomnia subgroups were identified, respectively. After the initial model comparison, LightGBM was selected for additional optimization. Bayesian optimization was conducted separately for the mild and severe insomnia subgroups within the development cohort, using 5-fold cross-validation and AUROC as the objective function. In addition, optimized classification thresholds were derived from the pooled out-of-fold predictions in the development cohort, with the Youden-index threshold used as the primary threshold and the maximum-F1 threshold used as a sensitivity analysis. The optimized hyperparameters, thresholds, and corresponding performance metrics are reported in Supplementary Tables 4, 5.

### 2.6 SHAP explainability analysis

To enhance model interpretability and clarify the relative contributions of each predictor variable, this study employed the SHapley Additive Explanations (SHAP) method to interpret the optimal models for the mild and severe insomnia subgroups. Based on a cooperative game theory framework, SHAP quantitatively assesses the impact of each feature on model predictions by calculating its SHAP value, thereby providing a transparent and intuitive explanation of the model. By comparing the distributions of SHAP scores across the two insomnia subgroup models, this study described the model-estimated contribution patterns of key variables in the identification of current cognitive impairment across different levels of sleep-duration-defined insomnia, along with their relative importance. Based on the full sample of the external validation cohort, SHAP feature importance plots, summary scatter plots, and dependency plots were generated to illustrate the specific roles of key variables in the direction and magnitude of model predictions. The SHAP analysis was used to provide exploratory information for risk stratification and referral prioritization, rather than to infer causal, mechanistic, or definitive biological differences between insomnia subgroups.

### 2.7 Development of an online assessment tool

To promote the clinical application of the models, this study developed online web applications for the mild and severe insomnia subgroups based on the optimal models identified through model comparison. After clinicians or users select the appropriate subgroup based on the severity of insomnia and enter relevant clinical parameters, the system automatically generates personalized probability estimates for cognitive impairment. The web application also provides visualizations of feature contributions, which may improve model interpretability and support clinical screening, assessment prioritization, and individualized follow-up planning.

### 2.8 Statistical analysis

Continuous variables are expressed as mean ± standard deviation, and categorical variables are expressed as frequency and percentage. Comparisons of baseline characteristics among the development cohort, temporal validation cohort, and external validation cohort were performed using one-way analysis of variance (ANOVA); Fisher’s exact test was used when the expected frequencies were small. Data preprocessing, feature selection, model development, and statistical analysis were all performed using R software (version 4.5.2). All tests were two-sided; a *p*-value of < 0.05 was considered statistically significant.

## 3 Results

### 3.1 Patient characteristics

As shown in Table 1, the mean ages of the three cohorts were 61.53, 60.82, and 63.64 years, respectively (*p* < 0.001), with the external validation cohort having the highest mean age. The proportion of females was 57.96, 56.09, and 59.80%, respectively (*p* = 0.09). The external validation cohort had a higher level of education (*p* < 0.001) and a higher proportion of urban residents and internet users (*p* < 0.001). Regarding physical and functional indicators, the three cohorts showed significant differences in the number of household members, BMI, waist circumference, number of chronic diseases, and ADL and IADL scores (all *p* < 0.001). The external validation cohort had the highest number of chronic diseases (3.75 ± 2.53), and both ADL and IADL scores were higher than those of the other two cohorts, indicating a relatively more severe degree of functional impairment. Regarding health-related characteristics, the external validation cohort had higher proportions of hypertension, dyslipidemia, diabetes, cancer, chronic lung disease, liver disease, heart disease, stroke, kidney disease, digestive system diseases, arthritis, and asthma, as well as higher rates of diabetes medication use, hypertension medication use, history of falls, and history of hip fractures compared to the other two cohorts (all *p* < 0.001). The proportion of participants reporting poor self-rated health, hearing, and vision was also relatively higher (all *p* < 0.001). Severe insomnia was present in 67.00% of the external validation cohort, which was higher than in the development cohort (54.76%) and the temporal validation cohort (54.81%) (*p* < 0.001). The sample sizes for model development, temporal validation, and external validation were 5,500, 4,231, and 500 cases, respectively, with 1,201, 885, and 160 cases of cognitive impairment, respectively; The prevalence of cognitive impairment was highest in the external validation cohort (32.00%), while the development and temporal validation cohorts had prevalences of 21.84 and 20.92%, respectively (*p* < 0.001).

**Table 1:** Baseline characteristics of participants in the development, temporal validation, and clinical external validation cohorts.

Variables	Development (CHARLS 2015, *n* = 5,500)	Temporal validation (CHARLS 2011, *n* = 4,231)	Clinical external validation (*n* = 500)	*p* value
Age (years), Mean ± SD	61.53 ± 9.80	60.82 ± 9.51	63.64 ± 7.63	<0.001
Number of family members, Mean ± SD	2.97 ± 1.31	3.58 ± 1.92	2.54 ± 1.33	<0.001
BMI, Mean ± SD	23.73 ± 3.58	23.10 ± 3.47	22.68 ± 2.82	<0.001
Waist circumference, Mean ± SD	86.07 ± 10.33	84.44 ± 10.10	83.96 ± 9.00	<0.001
Number of chronic conditions, Mean ± SD	2.33 ± 1.83	1.68 ± 1.52	3.75 ± 2.53	<0.001
ADL score, Mean ± SD	0.57 ± 1.17	0.52 ± 1.16	1.50 ± 1.92	<0.001
IADL score, Mean ± SD	0.59 ± 1.12	0.62 ± 1.19	1.53 ± 1.76	<0.001
Gender, *n* (%)				0.09
Female	3,188 (57.96%)	2,373 (56.09%)	299 (59.80%)	
Male	2,312 (42.04%)	1,858 (43.91%)	201 (40.20%)	
Education, *n* (%)				<0.001
Below high school	4,979 (90.53%)	3,889 (91.92%)	403 (80.60%)	
High school	450 (8.18%)	297 (7.02%)	47 (9.40%)	
College or above	71 (1.29%)	45 (1.06%)	50 (10.00%)	
Household registration, *n* (%)				<0.001
Rural	3,391 (61.65%)	2,695 (63.70%)	270 (54.00%)	
Urban	2,109 (38.35%)	1,536 (36.30%)	230 (46.00%)	
Marital status, *n* (%)				0.002
Other marital status	904 (16.44%)	677 (16.00%)	111 (22.20%)	
Married or partnered	4,596 (83.56%)	3,554 (84.00%)	389 (77.80%)	
Currently working, *n* (%)				<0.001
No	2,016 (37.40%)	1,688 (40.46%)	270 (54.00%)	
Yes	3,374 (62.60%)	2,484 (59.54%)	230 (46.00%)	
Living with children, *n* (%)				<0.001
No	2,493 (45.82%)	1,730 (41.91%)	333 (66.60%)	
Yes	2,948 (54.18%)	2,398 (58.09%)	167 (33.40%)	
Sociability, *n* (%)				0.043
No	2,971 (54.02%)	2,344 (55.43%)	297 (59.40%)	
Yes	2,529 (45.98%)	1,885 (44.57%)	203 (40.60%)	
Smoking status, *n* (%)				<0.001
No	4,058 (73.84%)	2,964 (70.05%)	389 (77.80%)	
Yes	1,438 (26.16%)	1,267 (29.95%)	111 (22.20%)	
Alcohol consumption, *n* (%)				<0.001
No	3,658 (66.53%)	2,938 (69.44%)	365 (73.00%)	
Yes	1,840 (33.47%)	1,293 (30.56%)	135 (27.00%)	
Internet use, *n* (%)				<0.001
No	5,238 (95.24%)	4,159 (98.34%)	417 (83.40%)	
Yes	262 (4.76%)	70 (1.66%)	83 (16.60%)	
Hypertension, *n* (%)				<0.001
No	3,077 (63.84%)	3,030 (72.01%)	241 (48.20%)	
Yes	1,743 (36.16%)	1,178 (27.99%)	259 (51.80%)	
Dyslipidemia, *n* (%)				<0.001
No	3,732 (79.54%)	3,698 (89.28%)	319 (63.80%)	
Yes	960 (20.46%)	444 (10.72%)	181 (36.20%)	
Diabetes, *n* (%)				<0.001
No	4,254 (88.53%)	3,914 (93.48%)	384 (76.80%)	
Yes	551 (11.47%)	273 (6.52%)	116 (23.20%)	
Cancer, *n* (%)				<0.001
No	4,738 (97.95%)	4,161 (98.86%)	477 (95.40%)	
Yes	99 (2.05%)	48 (1.14%)	23 (4.60%)	
Chronic lung disease, *n* (%)				<0.001
No	4,001 (82.67%)	3,678 (87.24%)	353 (70.60%)	
Yes	839 (17.33%)	538 (12.76%)	147 (29.40%)	
Liver disease, *n* (%)				<0.001
No	4,422 (91.78%)	4,006 (95.49%)	425 (85.00%)	
Yes	396 (8.22%)	189 (4.51%)	75 (15.00%)	
Heart disease, *n* (%)				<0.001
No	3,754 (77.98%)	3,576 (85.00%)	317 (63.40%)	
Yes	1,060 (22.02%)	631 (15.00%)	183 (36.60%)	
Stroke, *n* (%)				<0.001
No	4,648 (95.82%)	4,101 (97.09%)	430 (86.00%)	
Yes	203 (4.18%)	123 (2.91%)	70 (14.00%)	
Kidney disease, *n* (%)				<0.001
No	4,191 (86.97%)	3,888 (92.55%)	388 (77.60%)	
Yes	628 (13.03%)	313 (7.45%)	112 (22.40%)	
Digestive disease, *n* (%)				<0.001
No	2,965 (61.07%)	3,003 (71.18%)	262 (52.40%)	
Yes	1,890 (38.93%)	1,216 (28.82%)	238 (47.60%)	
Arthritis, *n* (%)				<0.001
No	2,274 (47.14%)	2,437 (57.79%)	203 (40.60%)	
Yes	2,550 (52.86%)	1,780 (42.21%)	297 (59.40%)	
Asthma, *n* (%)				<0.001
No	4,503 (92.98%)	3,972 (94.32%)	435 (87.00%)	
Yes	340 (7.02%)	239 (5.68%)	65 (13.00%)	
Diabetes medication use, *n* (%)				<0.001
No	4,390 (93.19%)	4,020 (96.01%)	422 (84.40%)	
Yes	321 (6.81%)	167 (3.99%)	78 (15.60%)	
Hypertension medication use, *n* (%)				<0.001
No	3,321 (70.80%)	3,307 (78.61%)	286 (57.20%)	
Yes	1,370 (29.20%)	900 (21.39%)	214 (42.80%)	
Falls, *n* (%)				<0.001
No	3,352 (60.95%)	3,330 (78.76%)	207 (41.40%)	
Yes	2,148 (39.05%)	898 (21.24%)	293 (58.60%)	
Hip fracture, *n* (%)				<0.001
No	5,249 (95.54%)	4,145 (98.04%)	468 (93.60%)	
Yes	245 (4.46%)	83 (1.96%)	32 (6.40%)	
Self−perceived health status, *n* (%)				<0.001
Poor	1,733 (31.54%)	1,614 (38.16%)	281 (56.20%)	
Average	2,832 (51.54%)	1,980 (46.81%)	162 (32.40%)	
Good	930 (16.92%)	636 (15.04%)	57 (11.40%)	
Hearing, *n* (%)				<0.001
Poor	887 (16.14%)	734 (17.36%)	120 (24.00%)	
Average	3,012 (54.80%)	1,829 (43.25%)	273 (54.60%)	
Good	1,597 (29.06%)	1,666 (39.39%)	107 (21.40%)	
Vision, *n* (%)				<0.001
Poor	1,389 (25.28%)	1,222 (28.90%)	159 (31.80%)	
Average	2,763 (50.28%)	1,862 (44.04%)	247 (49.40%)	
Good	1,343 (24.44%)	1,144 (27.06%)	94 (18.80%)	
Insomnia severity, *n* (%)				<0.001
Mild insomnia	2,488 (45.24%)	1,912 (45.19%)	165 (33.00%)	
Severe insomnia	3,012 (54.76%)	2,319 (54.81%)	335 (67.00%)	
Cognitive impairment, *n* (%)				<0.001
No	4,299 (78.16%)	3,346 (79.08%)	340 (68.00%)	
Yes	1,201 (21.84%)	885 (20.92%)	160 (32.00%)	

BMI, body mass index; ADL, activities of daily living; IADL, instrumental activities of daily living; SD, standard deviation; *n*, number of participants. Continuous variables are expressed as mean ± SD, and categorical variables are expressed as *n* (%). Percentages were calculated based on non-missing data for each variable; therefore, category totals may not sum to the total cohort size due to missing values. *p* values were calculated using one-way analysis of variance for continuous variables and Pearson’s chi-square test or Fisher’s exact test, as appropriate, for categorical variables. All tests compared the three cohorts overall and used non-missing values.

### 3.2 Model development and performance evaluation

**Figure 2:**
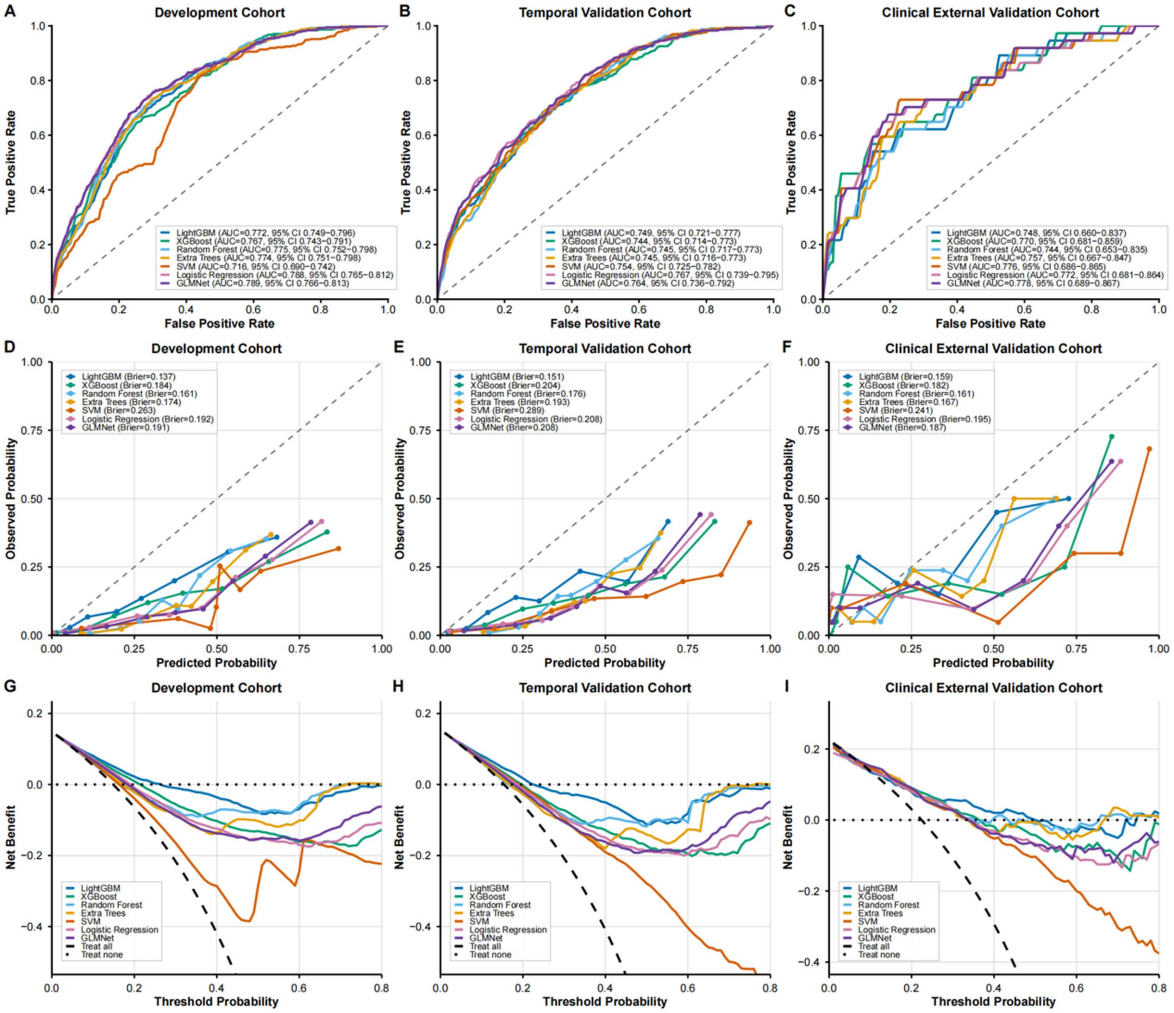
Performance of machine learning models for identifying cognitive impairment in the mild insomnia subgroup, including discrimination, calibration, and clinical utility. **(A–C)** Receiver operating characteristic (ROC) curves of the candidate models in the development, temporal validation, and clinical external validation cohorts, respectively. **(D–F)** Calibration curves of the candidate models in the development, temporal validation, and clinical external validation cohorts, respectively. **(G–I)** Decision curve analysis (DCA) curves of the candidate models in the development, temporal validation, and clinical external validation cohorts, respectively. Higher area under the ROC curve (AUROC) indicates better discrimination, and a lower Brier score indicates better calibration. The ROC legend reports AUROC values with 95% confidence intervals for each model.

**Figure 3:**
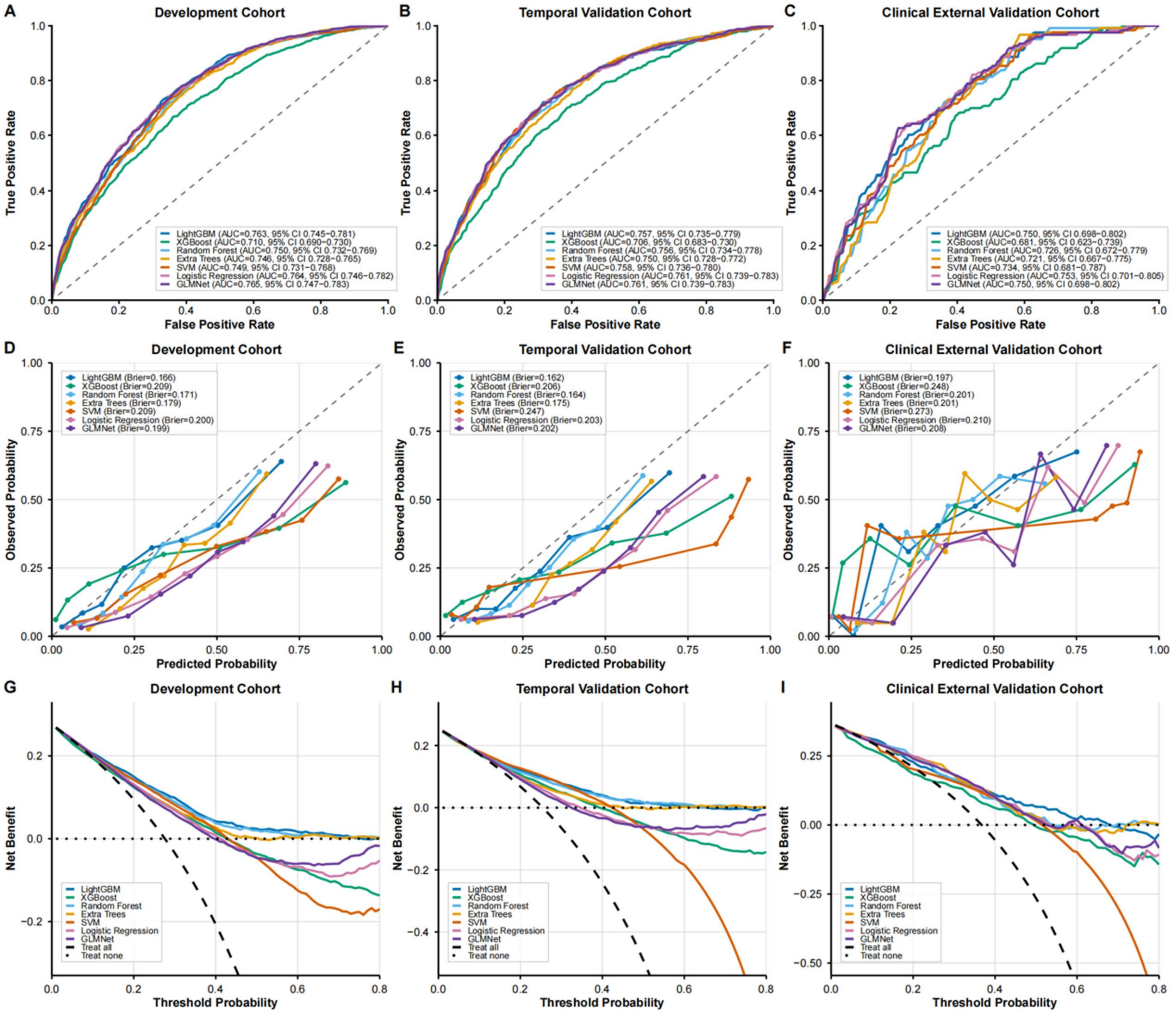
Performance of machine learning models for identifying cognitive impairment in the severe insomnia subgroup, including discrimination, calibration, and clinical utility. **(A–C)** Receiver operating characteristic (ROC) curves of the candidate models in the development, temporal validation, and clinical external validation cohorts, respectively. **(D–F)** Calibration curves of the candidate models in the development, temporal validation, and clinical external validation cohorts, respectively. **(G–I)** Decision curve analysis (DCA) curves of the candidate models in the development, temporal validation, and clinical external validation cohorts, respectively. Higher area under the ROC curve (AUROC) indicates better discrimination, and a lower Brier score indicates better calibration. The ROC legend reports AUROC values with 95% confidence intervals for each model.

**Figure 4:**
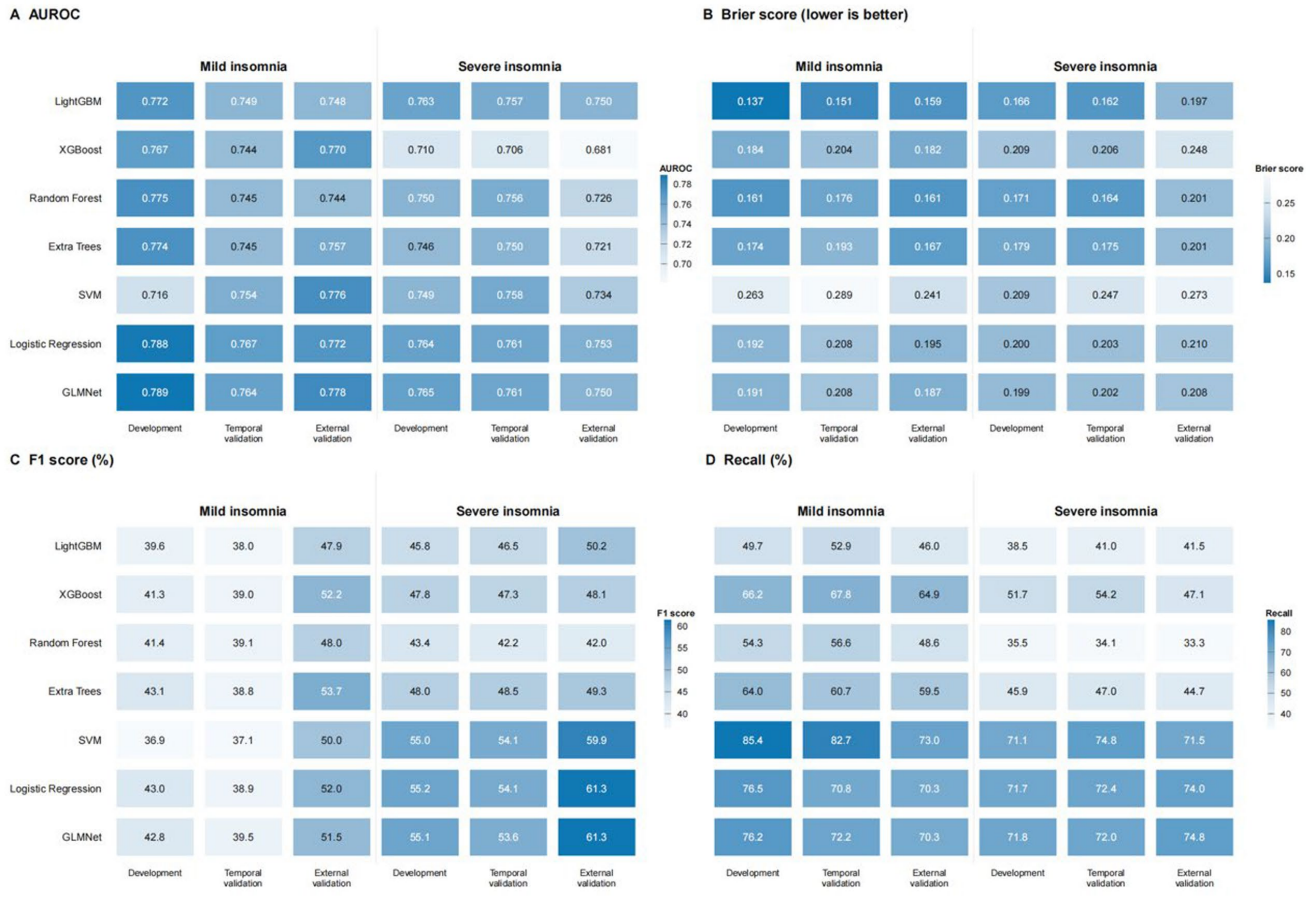
Heatmaps of candidate algorithm performance across insomnia subgroups and validation cohorts. **(A)** AUROC. **(B)** Brier score. **(C)** F1 score. **(D)** Recall. Each heatmap summarizes the performance of seven candidate algorithms in the mild and severe insomnia subgroups across the development, temporal validation, and clinical external validation cohorts. For AUROC, F1 score, and recall, darker colors indicate better performance; for Brier score, darker colors indicate lower values and better performance. F1 score and recall are presented as percentages.

After feature selection, this study developed seven candidate machine learning models for the mild and severe insomnia groups, respectively. The performance of each model across the three cohorts is shown in Figures 2, 3, Tables 2, 3, and visually summarized in Figure 4. Based on a comprehensive evaluation of discriminatory power, calibration, and clinical utility, LightGBM was selected as the final model. In the mild insomnia group, LightGBM achieved an accuracy of 77.45%, an AUROC of 0.772, and a Brier score of 0.137 on the development set. While GLMNet and LR had slightly higher AUROC values, their calibration performance was inferior to that of LightGBM. On the validation set, these three metrics were 73.33%, 0.749, and 0.151, respectively, and 77.58%, 0.748, and 0.159 in the external validation set. Although the AUROC of other models was comparable to that of LightGBM in some sets, LightGBM achieved the lowest Brier score across all three sets, with more balanced performance in terms of calibration curves and DCA, indicating relatively stable overall performance. In the severe insomnia group, LightGBM achieved an accuracy of 74.80%, an AUROC of 0.763, and a Brier score of 0.166 on the development set; on the temporal validation cohort, these figures were 75.98%, 0.757, and 0.162, respectively; and 69.85%, 0.750, and 0.197 in the external validation set. While LR and GLMNet achieved slightly higher or comparable AUROC values in some sets, LightGBM achieved the lowest Brier score across all three sets, demonstrating a more balanced combination of discriminative power, calibration, and clinical net benefit. LightGBM achieved the highest accuracy in the external validation set, further supporting its potential usefulness on independent data. Although LightGBM did not consistently outperform other models on every individual metric, it demonstrated more consistent calibration across the three cohorts and relatively stable clinical net benefit. Based on the combined results of ROC curves, calibration curves, and DCA analysis, LightGBM exhibited relatively stable generalization performance across different severity levels of insomnia and was selected as the final model for this study. The confusion matrices of the final LightGBM models under the default classification threshold of 0.5 in the development, temporal validation, and clinical external validation cohorts are presented in Supplementary Figure 4. As an additional optimization analysis, LightGBM was further tuned using Bayesian optimization within the development cohort. The Bayesian-optimized model showed comparable external validation AUROCs of 0.744 and 0.749 in the mild and severe insomnia subgroups, respectively. After applying optimized thresholds derived from the development cohort, threshold-dependent classification performance improved, especially in the severe insomnia subgroup, where the external validation F1 score increased from 0.521 to 0.612. In the mild insomnia subgroup, the external validation F1 score increased from 0.462 to 0.489 using the Youden-index threshold and to 0.494 using the maximum-F1 threshold. The optimized hyperparameters, thresholds, and detailed performance metrics are provided in Supplementary Tables 4, 5.

**Table 2:** Performance metrics of different machine learning models for identifying cognitive impairment in the mild insomnia subgroup across the development, temporal validation, and clinical external validation cohorts.

Dataset	Metric	LightGBM	XGBoost	Random forest	Extra trees	SVM	Logistic regression	GLMNet
Development cohort	Accuracy	77.45%	72.03%	77.09%	74.84%	56.47%	69.90%	69.73%
	Precision	32.92%	30.02%	33.39%	32.47%	23.49%	29.95%	29.78%
	Specificity	82.29%	73.04%	81.07%	76.72%	51.42%	68.74%	68.60%
	F1 score	39.61%	41.32%	41.36%	43.09%	36.85%	43.04%	42.82%
	Recall	49.73%	66.22%	54.32%	64.05%	85.41%	76.49%	76.22%
	AUROC	0.772	0.767	0.775	0.774	0.716	0.788	0.789
	Brier score	0.137	0.184	0.161	0.174	0.263	0.192	0.191
Temporal validation cohort	Accuracy	73.33%	67.31%	72.75%	70.45%	56.69%	65.69%	65.95%
	Precision	29.60%	27.40%	29.82%	28.50%	23.90%	26.83%	27.24%
	Specificity	77.06%	67.22%	75.70%	72.23%	51.95%	64.75%	64.81%
	F1 score	37.96%	39.02%	39.06%	38.79%	37.08%	38.92%	39.55%
	Recall	52.88%	67.80%	56.61%	60.68%	82.71%	70.85%	72.20%
	AUROC	0.749	0.744	0.745	0.745	0.754	0.767	0.764
	Brier score	0.151	0.204	0.176	0.193	0.289	0.208	0.208
External validation cohort	Accuracy	77.58%	73.33%	76.36%	76.97%	67.27%	70.91%	70.30%
	Precision	50.00%	43.64%	47.37%	48.89%	38.03%	41.27%	40.62%
	Specificity	86.72%	75.78%	84.38%	82.03%	65.62%	71.09%	70.31%
	F1 score	47.89%	52.17%	48.00%	53.66%	50.00%	52.00%	51.49%
	Recall	45.95%	64.86%	48.65%	59.46%	72.97%	70.27%	70.27%
	AUROC	0.748	0.770	0.744	0.757	0.776	0.772	0.778
	Brier score	0.159	0.182	0.161	0.167	0.241	0.195	0.187

This table summarizes the initial candidate-model comparison used for final model selection before the additional Bayesian optimization of LightGBM. Accuracy, precision, specificity, F1 score, and recall are presented as percentages. AUROC and Brier score are presented as decimals. Precision refers to positive predictive value, and recall refers to sensitivity. Higher AUROC indicates better discrimination, whereas a lower Brier score indicates better calibration.

**Table 3:** Performance metrics of different machine learning models for identifying cognitive impairment in the severe insomnia subgroup across the development, temporal validation, and clinical external validation cohorts.

Dataset	Metric	LightGBM	XGBoost	Random forest	Extra trees	SVM	Logistic regression	GLMNet
Development cohort	Accuracy	74.80%	68.76%	74.44%	72.61%	67.93%	67.83%	67.76%
	Precision	56.34%	44.33%	55.77%	50.40%	44.87%	44.81%	44.75%
	Specificity	88.63%	75.24%	89.27%	82.81%	66.71%	66.35%	66.21%
	F1 score	45.75%	47.75%	43.38%	48.02%	55.03%	55.16%	55.15%
	Recall	38.51%	51.74%	35.50%	45.85%	71.12%	71.72%	71.84%
	AUROC	0.763	0.710	0.750	0.746	0.749	0.764	0.765
	Brier score	0.166	0.209	0.171	0.179	0.209	0.200	0.199
Temporal validation cohort	Accuracy	75.98%	69.30%	76.28%	74.69%	67.70%	68.74%	68.26%
	Precision	53.66%	41.99%	55.52%	50.27%	42.36%	43.17%	42.67%
	Specificity	87.91%	74.44%	90.69%	84.15%	65.30%	67.50%	66.98%
	F1 score	46.49%	47.34%	42.23%	48.55%	54.08%	54.08%	53.59%
	Recall	41.02%	54.24%	34.07%	46.95%	74.75%	72.37%	72.03%
	AUROC	0.757	0.706	0.756	0.750	0.758	0.761	0.761
	Brier score	0.162	0.206	0.164	0.175	0.247	0.203	0.202
External validation cohort	Accuracy	69.85%	62.69%	66.27%	66.27%	64.78%	65.67%	65.37%
	Precision	63.75%	49.15%	56.94%	55.00%	51.46%	52.30%	51.98%
	Specificity	86.32%	71.70%	85.38%	78.77%	60.85%	60.85%	59.91%
	F1 score	50.25%	48.13%	42.05%	49.33%	59.86%	61.28%	61.33%
	Recall	41.46%	47.15%	33.33%	44.72%	71.54%	73.98%	74.80%
	AUROC	0.750	0.681	0.726	0.721	0.734	0.753	0.750
	Brier score	0.197	0.248	0.201	0.201	0.273	0.210	0.208

This table summarizes the initial candidate-model comparison used for final model selection before the additional Bayesian optimization of LightGBM. Accuracy, precision, specificity, F1 score, and recall are presented as percentages. AUROC and Brier score are presented as decimals. Precision refers to positive predictive value, and recall refers to sensitivity. Higher AUROC indicates better discrimination, whereas a lower Brier score indicates better calibration.

### 3.3 SHAP analysis of model interpretability

**Figure 5:**
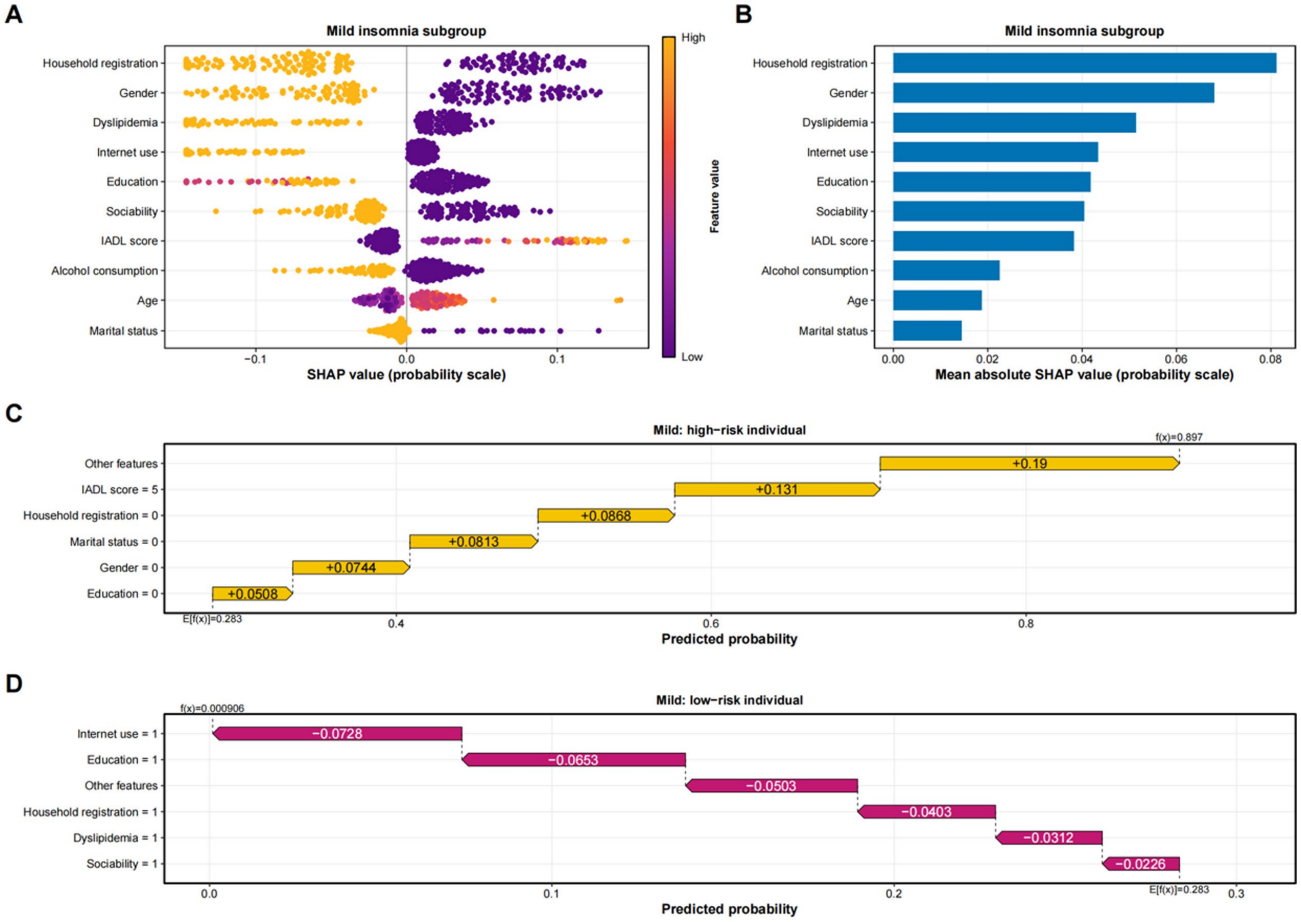
Global and local interpretability of the LightGBM model in the mild insomnia subgroup based on SHAP analysis. **(A)** SHAP summary dot plot showing the direction and magnitude of the contribution of each feature to individual predictions. Each dot represents one participant, with color indicating the corresponding feature value. **(B)** Horizontal bar plot showing the mean absolute SHAP value of each feature and its overall importance in the model. **(C)** Local SHAP waterfall plot for a representative high-risk individual. **(D)** Local SHAP waterfall plot for a representative low-risk individual. Features ranked higher in panels A and B contributed more strongly to the identification of cognitive impairment in the mild insomnia subgroup.

**Figure 6:**
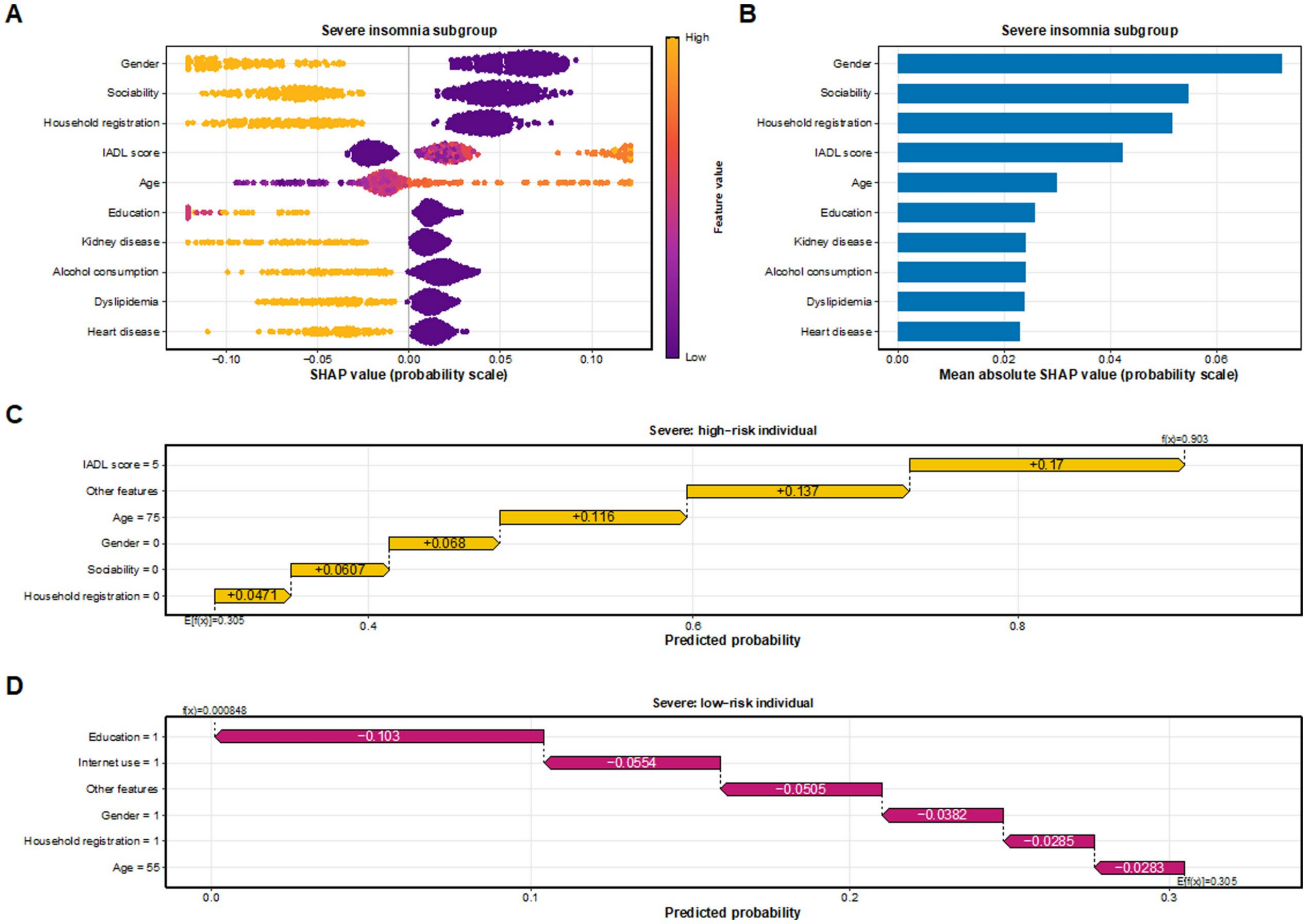
Global and local interpretability of the LightGBM model in the severe insomnia subgroup based on SHAP analysis. **(A)** SHAP summary dot plot showing the direction and magnitude of the contribution of each feature to individual predictions. Each dot represents one participant, with color indicating the corresponding feature value. **(B)** Horizontal bar plot showing the mean absolute SHAP value of each feature and its overall importance in the model. **(C)** Local SHAP waterfall plot for a representative high-risk individual. **(D)** Local SHAP waterfall plot for a representative low-risk individual. Features ranked higher in panels A and B contributed more strongly to the identification of cognitive impairment in the severe insomnia subgroup.

Figures 5, 6 present the SHAP interpretation results for the LightGBM model. In the mild insomnia group, household registration type, gender, dyslipidemia, internet use, educational level, social participation, IADL score, alcohol consumption, age, and marital status were the top 10 contributing features. Among these, household registration type, gender, and dyslipidemia had the highest SHA*p* values, indicating that sociodemographic factors, social participation, and functional status contributed relatively more to the model’s predictions. Local explanations reveal the feature contributions underlying individual predictions: In Figure 5C, the model-estimated probability for an individual with a higher predicted probability rose from the baseline of 0.283 to 0.897, with the main features contributing to a higher model-estimated probability being the combined contribution of other characteristics (+0.19), an IADL score of 5 (+0.131), rural household registration (+0.0868), unmarried status (+0.0813), female gender (+0.0744), and low educational attainment (+0.0508). In Figure 5D, the model-estimated probability for an individual with a lower predicted probability decreased from 0.283 to 0.0009, with the main features contributing to a lower model-estimated probability being internet use (−0.0728), high school education (−0.0653), the combined contribution of other characteristics (−0.0503), urban household registration (−0.0403), normal blood lipid levels (−0.0312), and social engagement (−0.0226). For the mild insomnia subgroup, Supplementary Figure 5 shows that age exhibits a nonlinear relationship with SHAP values, IADL scores show a gradient relationship, educational level follows a stratified distribution, and household registration type, gender, dyslipidemia, internet use, social engagement, and alcohol consumption show discrete distribution patterns. In the severe insomnia group, gender, social engagement, household registration type, IADL scores, age, educational level, kidney disease, alcohol consumption, dyslipidemia, and heart disease were the top 10 contributing features, with gender, social engagement, and household registration type showing the highest SHAP values. Compared with the mild insomnia group, social behavioral factors and certain chronic diseases showed more prominent SHAP contributions. In Figure 6C, the model-estimated probability for an individual with a higher predicted probability increased from the baseline of 0.305 to 0.903. The main features contributing to a higher model-estimated probability were an IADL score of 5 (+0.17), the combined contribution of other characteristics (+0.137), age 75 (+0.116), female gender (+0.068), lack of social engagement (+0.0607), and rural residency (+0.0471). In Figure 6D, the model-estimated probability for an individual with a lower predicted probability decreased from 0.305 to 0.0008. The main features contributing to a lower model-estimated probability were high school education (−0.103), internet use (−0.0554), the combined contribution of other characteristics (−0.0505), male gender (−0.0382), urban household registration (−0.0285), and younger age (−0.0283). Supplementary Figure 6 further shows that, in the severe insomnia group, age and IADL score exhibit nonlinear or hierarchical associations with SHAP values, whereas gender, social participation, household registration type, kidney disease, alcohol consumption, and dyslipidemia show discrete distribution patterns. In summary, the SHAP analysis indicates that demographic and sociological characteristics, factors related to social participation, and indicators of functional impairment contributed to model predictions in both subgroups; however, their relative importance and specific contribution patterns were not consistent. This suggests that cognitive impairment identification in individuals with different degrees of insomnia severity may be associated with different cross-sectional feature contribution patterns.

### 3.4 Development of an online assessment tool

Based on the final LightGBM model, this study developed two online calculators for assessing the risk of cognitive impairment. The calculator for the mild insomnia group can be accessed at: https://mild-insomnia-cognition-risk.shinyapps.io/MildInsomniaCognitionCalculator/, and the calculator for the severe insomnia group can be accessed at: https://severe-insomnia-cognition-risk.shinyapps.io/SevereInsomniaCognitionCalculator/. After users enter relevant clinical information, the system automatically generates personalized predictions of the probability of cognitive impairment, risk stratification results, and corresponding clinical recommendations. The interfaces of the two online calculators are shown in Supplementary Figures 7, 8, respectively.

## 4 Discussion

In this study, we developed cognitive impairment identification models for populations with mild and severe insomnia, respectively, and evaluated the models’ generalization performance in a cross-wave temporal validation cohort and a clinical external validation cohort. Because predictors and outcomes were measured within the same survey wave, the models should be interpreted as tools for cross-sectional risk identification and auxiliary screening of current cognitive impairment, rather than as longitudinal prognostic models for predicting incident cognitive impairment. We also used the SHAP method to interpret the basis for the models’ predictions. The results showed that the model maintained moderate and relatively stable discriminatory ability, acceptable calibration, and potential clinical net benefit in both validation scenarios, suggesting that machine learning models based on standard demographic characteristics, functional status, lifestyle, and chronic disease information may provide auxiliary information for preliminary identification of current cognitive impairment risk and risk stratification in individuals with insomnia. SHAP analysis indicated that the feature contribution patterns for individuals with mild insomnia were not entirely consistent with those for individuals with severe insomnia, suggesting that cognitive impairment identification among individuals with insomnia may show heterogeneity across different severity levels. These findings provide a basis for understanding the model’s cross-sectional contribution patterns and may offer guidance for auxiliary risk stratification and referral prioritization for formal cognitive assessment in sleep medicine practice.

### 4.1 Stratified risk prediction via interpretable ensemble learning

LightGBM demonstrated consistent overall performance in both the mild insomnia and severe insomnia subgroups. The model exhibited moderate discriminatory performance across the development, validation, and external clinical validation cohorts, and achieved the lowest Brier score in all three cohorts of both subgroups, indicating relatively favorable calibration rather than uniformly superior predictive performance. Specifically, in the mild insomnia group, LightGBM achieved AUROCs of 0.772, 0.749, and 0.748 across the three cohorts; in the severe insomnia group, the corresponding values were 0.763, 0.757, and 0.750. Although the AUROC values for Logistic regression or GLMNet were similar to or even slightly higher than those of LightGBM in some cohorts, for the stratified assessment of cognitive impairment risk in individuals with insomnia, what holds greater clinical value is not the optimal fit achieved on a single dataset, but rather a model that maintains a balanced profile of discrimination, calibration, and clinical utility across different time points and populations. Even if the improvement in discriminatory power is limited, more consistent calibration may support more cautious risk communication and preliminary risk stratification (Van Calster et al., 2019; Riley et al., 2024). In an additional optimization analysis, we further conducted Bayesian optimization for the selected LightGBM model. The Bayesian-optimized model showed comparable external validation AUROCs to the original LightGBM model, with AUROCs of 0.744 and 0.749 in the mild and severe insomnia subgroups, respectively. Although Bayesian optimization did not substantially increase external AUROC, the optimized-threshold analysis improved threshold-dependent classification metrics, especially in the severe insomnia subgroup, where the external validation F1 score increased from 0.521 under the default 0.5 threshold to 0.612 under the optimized threshold. These findings suggest that, in imbalanced clinical screening scenarios, threshold selection may be as important as model selection when the goal is to improve sensitivity and F1 score.

LightGBM, an ensemble learning framework based on gradient boosting, is capable of capturing the underlying nonlinear relationships and interactions among demographic and sociological factors, social engagement, functional status, and the burden of chronic diseases (Ke et al., 2017; Zhang et al., 2019). This capability is particularly important for assessing cognitive risks associated with insomnia. Mild insomnia and severe insomnia differ in self-reported nightly sleep duration and may also be associated with different functional profiles and comorbid burden; therefore, the cross-sectional predictor patterns related to cognitive impairment identification may also differ (Benjamins et al., 2017). Combined with SHAP interpretability analysis, this study not only generates personalized risk probabilities but also identifies the key factors driving an increase or decrease in risk (Lundberg and Lee, 2017). By leveraging an online risk calculator, this framework transforms machine learning models from relatively “black box” predictive tools into auxiliary assessment tools that are clinically understandable and applicable. Compared to traditional screening methods that provide only a single risk score, this strategy—which combines predictive performance with interpretability—is more likely to be applied in settings such as sleep clinics, primary care, and community screening.

### 4.2 Shared influencing factors

In both the mild insomnia group and the severe insomnia group, age, gender, educational level, IADL scores, social engagement, household registration status, and internet use all demonstrated consistent patterns of influence and significance, suggesting that these factors may constitute a common basis for identifying the risk of cognitive impairment among individuals with insomnia. Specifically, advancing age and female gender were associated with a higher risk of cognitive impairment in both subgroups; conversely, higher educational attainment, lower levels of impairment in Instrumental Activities of Daily Living (IADL), more active social engagement, urban residency, and internet use were associated with a lower risk of cognitive impairment. These findings are consistent with existing research on cognitive aging, gender differences, and cognitive reserve, which indicates that advanced age is typically associated with an increased risk of cognitive decline (Lipnicki et al., 2017), while educational attainment, functional independence, and greater access to social and informational resources contribute to maintaining cognitive health (Sharp and Gatz, 2011; Raimo et al., 2024). In both insomnia subgroups, the IADL score showed a consistent contribution to the model-estimated probability of cognitive impairment. This finding should be interpreted cautiously because IADL reflects functional status and may partly overlap with functional consequences associated with cognitive impairment. Therefore, IADL should not be interpreted as an independent causal or temporally antecedent predictor, but rather as a routinely available functional correlate that may help identify individuals who require further formal cognitive assessment.

These findings suggest that the risk of cognitive impairment among individuals with insomnia is not driven by a single sleep problem or a single risk factor, but is more likely the result of the combined effects of multiple biological, functional, and sociobehavioral factors (Sexton et al., 2020). Previous studies have shown that a higher level of education can, to some extent, mitigate the impact of neurodegenerative changes on clinical manifestations, while impairment in IADLs is often regarded as an important early indicator of cognitive decline (Meng and D'Arcy, 2012; Jekel et al., 2015). In this study, educational attainment and IADL scores remained highly significant in both insomnia subgroups, further supporting this view. At the same time, social engagement and internet use were associated with a lower risk of cognitive impairment, consistent with previous observations that social connections, informational stimulation, and cognitive engagement contribute to maintaining cognitive health in older adults (Kelly et al., 2017; Yu et al., 2022). For individuals with insomnia, these factors may be particularly important, as insomnia not only affects nighttime sleep itself but may also indirectly increase cognitive vulnerability through daytime fatigue, reduced activity, and social withdrawal (Sexton et al., 2020; Krystal, 2007). Therefore, even against the adverse backdrop of insomnia, greater cognitive reserve, better functional status, and more extensive social resources may still reduce the risk of cognitive impairment to some extent.

In addition, alcohol consumption and dyslipidemia exhibited a certain degree of negative SHAP contribution in both subgroups; however, this finding is more likely to reflect the combined influence of multiple factors—including the level of health management, treatment exposure, access to medical care, differences in behavioral patterns, and residual confounders—and should not be directly interpreted as indicating a protective effect in and of itself (Shrank et al., 2011; Liang and Chikritzhs, 2013). Therefore, while these variables may help distinguish model-estimated probabilities in the present model, their biological interpretation and clinical implications should be approached with caution.

### 4.3 Differential predictor patterns across insomnia severity

This study also found that the patterns driving the risk of cognitive impairment vary significantly across different levels of insomnia severity. In the mild insomnia group, sociodemographic and sociobehavioral factors—such as household registration status, internet use, educational attainment, and marital status—exhibited a stronger predictive role, suggesting that the risk of cognitive impairment at this stage is more strongly influenced by social resources, access to information, and the level of family support. Rural household registration, lower educational attainment, lack of internet use, and being unmarried or without a partner are all associated with a higher risk of cognitive impairment. Previous studies have shown that rural household registration is associated with poorer cognitive function, and that social support from friends can mitigate this adverse effect to some extent (Peng et al., 2024); internet use is associated with better cognitive function and slower cognitive decline (Yu et al., 2022); and there is also an association between marital status and cognitive impairment, with social support likely playing an important role (Zhang et al., 2024). Furthermore, social activities, social networks, and social support generally contribute to maintaining cognitive health in older adults (Kelly et al., 2017). Based on these findings, we hypothesize that during the early stages of insomnia, the risk of cognitive impairment may stem from the combined effects of sleep problems and sociobehavioral factors, rather than being a direct reflection of reduced sleep duration alone. These factors are particularly important for individuals with insomnia, as insomnia not only affects nighttime sleep but is also associated with cognitive aging and impaired daytime functioning; against a backdrop of limited social resources and reduced cognitive stimulation, their cognitive vulnerability may be further exacerbated (Sexton et al., 2020; Krystal, 2007).

In the severe insomnia group, aside from age and gender, social engagement, IADL scores, and indicators of chronic conditions such as kidney disease and heart disease were more strongly associated with the model-estimated probability of cognitive impairment. This pattern may reflect the greater clinical complexity of individuals with very short sleep duration, but it should not be interpreted as evidence that functional impairment temporally precedes or causes cognitive impairment. Previous studies have shown that greater severity of insomnia is associated with poorer cognitive performance and more pronounced impairment in daytime functioning (Baril et al., 2022). At the same time, heart disease and kidney disease have been associated with cognitive impairment in previous studies, possibly reflecting vascular, metabolic, and inflammatory pathways (Zuo and Wu, 2022; Zammit et al., 2016). Therefore, among individuals with severe insomnia, cognitive risk is not only associated with sleep problems themselves but may also reflect the combined effects of severe sleep deprivation, functional decline, and comorbid chronic conditions.

Based on these findings, the identification of current cognitive impairment in individuals with mild insomnia and those with severe insomnia was informed by different model-estimated feature contribution patterns, rather than by a single uniform set of predictors. In the mild insomnia subgroup, sociodemographic and behavioral characteristics, such as educational attainment, social engagement, internet use, and household registration, showed relatively greater contributions to the model-estimated probability of cognitive impairment. In the severe insomnia subgroup, functional status and chronic disease-related variables, including IADL score, heart disease, and kidney disease, appeared to contribute more prominently to model predictions. These findings suggest that a one-size-fits-all assessment framework may not fully capture the heterogeneity of current cognitive impairment identification across different levels of sleep-duration-defined insomnia. However, these subgroup differences should be interpreted as exploratory predictive patterns rather than causal, mechanistic, or definitive biological differences. Therefore, subgroup-aware assessment and follow-up prioritization may be considered in clinical screening settings, while formal cognitive assessment remains necessary for diagnostic confirmation.

### 4.4 Clinical implications

This study constructed and validated cognitive impairment identification models for both the mild insomnia group and the severe insomnia group, and developed an online risk calculator, thereby enhancing the models’ potential auxiliary value for clinical translation. By integrating routine clinical interviews and basic health assessment data, this tool may support preliminary risk stratification and screening for cognitive impairment in individuals with insomnia within sleep clinics, geriatric medicine clinics, community health service centers, and primary care follow-up settings. It requires no additional laboratory tests or complex specialized examinations, offering reasonable accessibility for preliminary assessment. For individuals identified by the model as having a higher model-estimated probability of cognitive impairment, further steps such as more systematic cognitive assessments, referrals to specialists, or enhanced follow-up can be arranged to improve the efficiency of limited healthcare resources. The intended clinical role of this tool is not to replace standard cognitive screening tests, but to help prioritize individuals for formal cognitive assessment when comprehensive cognitive testing is not immediately available. In this sense, the model may serve as a referral-prioritization and preliminary triage tool in community, primary-care, geriatric, and sleep-medicine settings. Importantly, the tool is intended for short-sleeping individuals and should not be used to compare cognitive impairment risk between normal sleepers and individuals with sleep-duration-defined insomnia. However, given the moderate external validation performance, the model should not be used as a standalone diagnostic instrument. In practical application, if key input variables are missing, significantly abnormal, or of unreliable quality, the model’s output should be interpreted with caution, and it is recommended to supplement or verify the relevant information before proceeding with risk assessment. This tool is primarily intended for use by clinicians and primary care workers who have received training in basic geriatric health assessment, and is designed to supplement, not replace, formal cognitive assessments and clinical judgment.

The SHAP method enables the model to estimate the probability of current cognitive impairment and to show which features contribute to the model-estimated probability for a given individual. This may help healthcare professionals identify key contributors to individual-level model output, such as functional status indicators, social engagement, access to information, and chronic disease burden, thereby providing auxiliary information for sleep management, health education, and comprehensive assessment. The results should be interpreted together with standardized cognitive assessment, clinical history, and professional judgment, and should not be used as a substitute for formal neuropsychological assessment or clinical diagnosis.

### 4.5 Limitations

This study has several limitations. First, both the development and temporal validation cohorts were derived from CHARLS; therefore, the CHARLS 2011 validation should be interpreted as a cross-wave transportability assessment rather than a prospective prediction of future cognitive impairment. The clinical external validation cohort was from a single center with a relatively limited sample size, and the external validation performance remained moderate; therefore, the model should be regarded as an auxiliary screening and preliminary risk-stratification tool rather than a standalone diagnostic instrument. In addition, this study did not construct a long-term longitudinal prediction model using all available CHARLS waves or validate the model in another national cohort, such as HRS or CLHLS. Thus, the present models should not be interpreted as tools for predicting incident cognitive impairment over long-term follow-up. Second, several key variables, including sleep duration, were based on self-report and may be affected by recall or reporting bias. The mild and severe insomnia subgroups were defined according to self-reported sleep duration rather than formal clinical insomnia diagnoses, and detailed sleep phenotypes or objective sleep-monitoring data were unavailable. Because sleep duration is a continuous variable and the clinical external validation cohort did not include normal sleepers, the present models should not be used to estimate cognitive impairment risk relative to normal sleepers. Third, this was a cross-sectional risk-identification study. Predictors and outcomes were measured within the same survey wave, and ADL and IADL scores may partly overlap with functional consequences of cognitive impairment. Therefore, the identified associations should not be interpreted as causal effects, and the model should supplement, not replace, standardized cognitive assessment and clinical judgment. Future studies should validate this framework using standardized insomnia assessments, objective sleep measures, formal neuropsychological diagnoses, multi-wave longitudinal follow-up, independent national cohorts, and appropriate methods for handling attrition and informative censoring, such as IPW or longitudinal multiple imputation.

## 5 Conclusion

This study developed and validated cognitive impairment identification models for populations with mild and severe insomnia. LightGBM demonstrated relatively stable discriminatory ability, calibration performance, and clinical net benefit across the development, cross-wave temporal validation, and clinical external validation cohorts, although its external validation performance remained moderate. SHAP analysis showed that feature contribution patterns differed by insomnia severity: in individuals with mild insomnia, model-estimated probability was more strongly associated with sociodemographic and sociobehavioral factors, whereas in those with severe insomnia, it was more strongly associated with functional status indicators and chronic disease burden. In conjunction with an online risk calculator, this study provides an interpretable auxiliary tool for preliminary risk stratification and referral prioritization for formal cognitive assessment among middle-aged and older adults with insomnia. This tool should be used together with formal cognitive assessment and clinical judgment rather than as a standalone diagnostic instrument or a tool for predicting future incident cognitive impairment. Future prospective, multicenter studies are needed to validate its stability, reproducibility, and clinical utility in real-world settings.

## Data Availability

The CHARLS datasets analyzed in this study are available through the official China Health and Retirement Longitudinal Study platform upon registration and approval of a data-use application. The de-identified clinical data are available from the corresponding authors upon reasonable request.
