# Supplementary material for: Interpretable machine learning models for identifying cognitive impairment in middle-aged and older adults with mild and severe insomnia: development, temporal validation, and clinical external validation
Source: Front Aging Neurosci. 2026 Jun 23;18:1855864. doi: 10.3389/fnagi.2026.1855864 (PMC13337873; doi:10.3389/fnagi.2026.1855864)
Supplement: Supplementary file 1 [file Data_Sheet_1.docx]

**Supplementary Materials**

**Supplementary Table 1.** Definitions and coding of study variables.

| **Variable** | **Definition and coding** |
| --- | --- |
| **Outcome variable** | |
| Cognitive impairment | Binary outcome variable. Defined according to the global cognitive score. Participants with a global cognitive score < 6 were classified as having cognitive impairment (Yes = 1), and those with a score ≥ 6 were classified as not having cognitive impairment (No = 0). |
| Global cognitive score | Continuous variable ranging from 0 to 21, representing global cognitive function. It was calculated as the sum of episodic memory and executive function scores. Episodic memory was assessed as the average of immediate and delayed word recall scores (0–10). Executive function was assessed using the TICS-10 and figure drawing tasks (0–11). Higher scores indicate better cognitive function. |
| **Subgroup definition variable** | |
| Insomnia severity | Variable used for participant selection and subgroup classification rather than as a predictor in model analysis. Mild insomnia was defined as 5 h ≤ sleep duration < 6 h, and severe insomnia was defined as sleep duration < 5 h. |
| **Continuous candidate predictors** | |
| Age | Continuous variable in years. |
| Number of family members | Continuous variable indicating the number of family members in the household. |
| BMI | Continuous variable representing body mass index, calculated as weight in kilograms divided by height in meters squared (kg/m²). |
| Waist circumference | Continuous variable in centimeters (cm). |
| Number of chronic conditions | Continuous variable representing the total number of self-reported doctor-diagnosed chronic conditions. |
| ADL score | Continuous variable ranging from 0 to 6, calculated as the sum of six activities of daily living (dressing, bathing, eating, getting in/out of bed, toileting, and continence). Each item was coded as 0/1 according to whether difficulty was present. Higher scores indicate poorer functional status. |
| IADL score | Continuous variable ranging from 0 to 5, calculated as the sum of five instrumental activities of daily living (doing household chores, cooking, shopping, making phone calls, and managing finances). Each item was coded as 0/1 according to whether difficulty was present. Higher scores indicate poorer functional status. |
| **Categorical candidate predictors** | |
| Gender | Binary variable: 0 = Female, 1 = Male. |
| Education | Ordinal variable: 0 = Below high school, 1 = High school, 2 = College or above. |
| Household registration | Binary variable: 0 = Rural, 1 = Urban. |
| Marital status | Binary variable: 0 = Other marital status (separated, divorced, widowed, or never married), 1 = Married or partnered. |
| Currently working | Binary variable: 0 = No, 1 = Yes. |
| Living with children | Binary variable: 0 = No, 1 = Yes. |
| Sociability | Binary variable indicating whether the participant engaged in social activities regularly: 0 = No, 1 = Yes. |
| Smoking status | Binary variable: 0 = No, 1 = Yes (current smoking). |
| Alcohol consumption | Binary variable: 0 = No, 1 = Yes (current drinking). |
| Internet use | Binary variable: 0 = No, 1 = Yes. |
| Life satisfaction | Ordinal variable based on self-reported life satisfaction: 1 = Not satisfied, 2 = Average, 3 = Satisfied. |
| Self-perceived health status | Ordinal variable: 1 = Poor, 2 = Average, 3 = Good. |
| Hearing | Ordinal variable: 1 = Poor, 2 = Average, 3 = Good. |
| Vision | Ordinal variable: 1 = Poor, 2 = Average, 3 = Good. |
| Hypertension | Binary variable indicating self-reported doctor-diagnosed hypertension: 0 = No, 1 = Yes. |
| Dyslipidemia | Binary variable indicating self-reported doctor-diagnosed dyslipidemia: 0 = No, 1 = Yes. |
| Diabetes | Binary variable indicating self-reported doctor-diagnosed diabetes: 0 = No, 1 = Yes. |
| Cancer | Binary variable indicating self-reported doctor-diagnosed cancer: 0 = No, 1 = Yes. |
| Chronic lung disease | Binary variable indicating self-reported doctor-diagnosed chronic lung disease: 0 = No, 1 = Yes. |
| Liver disease | Binary variable indicating self-reported doctor-diagnosed liver disease: 0 = No, 1 = Yes. |
| Heart disease | Binary variable indicating self-reported doctor-diagnosed heart disease: 0 = No, 1 = Yes. |
| Stroke | Binary variable indicating self-reported doctor-diagnosed stroke: 0 = No, 1 = Yes. |
| Kidney disease | Binary variable indicating self-reported doctor-diagnosed kidney disease: 0 = No, 1 = Yes. |
| Digestive disease | Binary variable indicating self-reported doctor-diagnosed digestive disease: 0 = No, 1 = Yes. |
| Arthritis | Binary variable indicating self-reported doctor-diagnosed arthritis: 0 = No, 1 = Yes. |
| Asthma | Binary variable indicating self-reported doctor-diagnosed asthma: 0 = No, 1 = Yes. |
| Falls | Binary variable: 0 = No, 1 = Yes. |
| Hip fracture | Binary variable: 0 = No, 1 = Yes. |
| Diabetes medication use | Binary variable: 0 = No, 1 = Yes. |
| Hypertension medication use | Binary variable: 0 = No, 1 = Yes. |

Outcome, subgroup definition, and candidate predictors included in the study are presented. Cognitive impairment was defined according to the global cognitive score derived from episodic memory and executive function. Mild insomnia was defined as 5 h ≤ sleep duration < 6 h, and severe insomnia was defined as sleep duration < 5 h.

**Supplementary Table 2.** Variance inflation factor (VIF) values of predictors included in the mild insomnia subgroup.

| **Feature** | **VIF** |
| --- | --- |
| IADL score | 1.30 |
| ADL score | 1.29 |
| Gender | 1.16 |
| Alcohol consumption | 1.16 |
| Internet use | 1.11 |
| Age | 1.09 |
| Marital status | 1.06 |
| Household registration | 1.05 |
| Education | 1.05 |
| Sociability | 1.04 |
| Dyslipidemia | 1.03 |

**Supplementary Table 3.** Variance inflation factor (VIF) values of predictors included in the severe insomnia subgroup.

| **Feature** | **VIF** |
| --- | --- |
| IADL score | 1.32 |
| ADL score | 1.32 |
| Gender | 1.14 |
| Alcohol consumption | 1.12 |
| Age | 1.10 |
| Heart disease | 1.07 |
| Self-perceived health status | 1.07 |
| Internet use | 1.07 |
| Dyslipidemia | 1.06 |
| Marital status | 1.06 |
| Household registration | 1.04 |
| Kidney disease | 1.04 |
| Waist circumference | 1.04 |
| Education | 1.04 |
| Falls | 1.03 |
| Vision | 1.03 |
| Sociability | 1.02 |

**Supplementary Table 4.** Optimized hyperparameters and classification thresholds of the Bayesian-optimized LightGBM models.

| **Subgroup** | **Trees** | **Tree depth** | **Minimum node size** | **Sample size** | **mtry** | **Learning rate** | **Youden-index threshold** | **Maximum-F1 threshold** |
| --- | --- | --- | --- | --- | --- | --- | --- | --- |
| Mild insomnia | 595 | 2 | 37 | 0.936 | 1 | 0.026 | 0.348 | 0.484 |
| Severe insomnia | 735 | 2 | 9 | 0.661 | 6 | 0.021 | 0.304 | 0.304 |

Bayesian optimization was conducted within the development cohort. The optimized thresholds were derived from pooled out-of-fold predictions in the development cohort and then fixed for temporal and clinical external validation cohorts. mtry indicates the number of randomly selected predictors considered at each split.

**Supplementary Table 5.** Performance of the Bayesian-optimized LightGBM model under default and optimized thresholds.

**Panel A.** Mild insomnia subgroup.

| **Cohort (n/events)** | **Threshold type** | **Threshold** | **AUROC (95% CI)** | **AUPRC** | **Brier score** | **Accuracy** | **Precision** | **Sensitivity** | **Specificity** | **F1 score** | **Calibration intercept** | **Calibration slope** |
| --- | --- | --- | --- | --- | --- | --- | --- | --- | --- | --- | --- | --- |
| Development cohort (n=2488; events=370) | Default | 0.500 | 0.779 (0.755-0.802) | 0.383 | 0.152 | 0.764 | 0.336 | 0.600 | 0.793 | 0.431 | -1.276 | 0.880 |
| Development cohort (n=2488; events=370) | Youden index | 0.348 | 0.779 (0.755-0.802) | 0.383 | 0.152 | 0.685 | 0.290 | 0.773 | 0.669 | 0.422 | -1.276 | 0.880 |
| Development cohort (n=2488; events=370) | Maximum F1 | 0.484 | 0.779 (0.755-0.802) | 0.383 | 0.152 | 0.759 | 0.336 | 0.635 | 0.780 | 0.439 | -1.276 | 0.880 |
| Temporal validation cohort (n=1912; events=295) | Default | 0.500 | 0.749 (0.721-0.778) | 0.359 | 0.169 | 0.727 | 0.304 | 0.597 | 0.751 | 0.403 | -1.360 | 0.810 |
| Temporal validation cohort (n=1912; events=295) | Youden index | 0.348 | 0.749 (0.721-0.778) | 0.359 | 0.169 | 0.630 | 0.256 | 0.732 | 0.612 | 0.379 | -1.360 | 0.810 |
| Temporal validation cohort (n=1912; events=295) | Maximum F1 | 0.484 | 0.749 (0.721-0.778) | 0.359 | 0.169 | 0.722 | 0.304 | 0.620 | 0.741 | 0.408 | -1.360 | 0.810 |
| Clinical external validation cohort (n=165; events=37) | Default | 0.500 | 0.744 (0.655-0.833) | 0.495 | 0.166 | 0.745 | 0.439 | 0.486 | 0.820 | 0.462 | -0.451 | 0.503 |
| Clinical external validation cohort (n=165; events=37) | Youden index | 0.348 | 0.744 (0.655-0.833) | 0.495 | 0.166 | 0.709 | 0.404 | 0.622 | 0.734 | 0.489 | -0.451 | 0.503 |
| Clinical external validation cohort (n=165; events=37) | Maximum F1 | 0.484 | 0.744 (0.655-0.833) | 0.495 | 0.166 | 0.752 | 0.455 | 0.541 | 0.812 | 0.494 | -0.451 | 0.503 |

**Panel B.** Severe insomnia subgroup.

| **Cohort (n/events)** | **Threshold type** | **Threshold** | **AUROC (95% CI)** | **AUPRC** | **Brier score** | **Accuracy** | **Precision** | **Sensitivity** | **Specificity** | **F1 score** | **Calibration intercept** | **Calibration slope** |
| --- | --- | --- | --- | --- | --- | --- | --- | --- | --- | --- | --- | --- |
| Development cohort (n=3012; events=831) | Default | 0.500 | 0.764 (0.746-0.782) | 0.558 | 0.167 | 0.748 | 0.556 | 0.433 | 0.868 | 0.487 | -0.269 | 0.965 |
| Development cohort (n=3012; events=831) | Youden index | 0.304 | 0.764 (0.746-0.782) | 0.558 | 0.167 | 0.669 | 0.442 | 0.768 | 0.631 | 0.561 | -0.269 | 0.965 |
| Development cohort (n=3012; events=831) | Maximum F1 | 0.304 | 0.764 (0.746-0.782) | 0.558 | 0.167 | 0.669 | 0.442 | 0.768 | 0.631 | 0.561 | -0.269 | 0.965 |
| Temporal validation cohort (n=2319; events=590) | Default | 0.500 | 0.759 (0.737-0.781) | 0.530 | 0.164 | 0.752 | 0.515 | 0.451 | 0.855 | 0.481 | -0.427 | 0.943 |
| Temporal validation cohort (n=2319; events=590) | Youden index | 0.304 | 0.759 (0.737-0.781) | 0.530 | 0.164 | 0.655 | 0.405 | 0.764 | 0.617 | 0.530 | -0.427 | 0.943 |
| Temporal validation cohort (n=2319; events=590) | Maximum F1 | 0.304 | 0.759 (0.737-0.781) | 0.530 | 0.164 | 0.655 | 0.405 | 0.764 | 0.617 | 0.530 | -0.427 | 0.943 |
| Clinical external validation cohort (n=335; events=123) | Default | 0.500 | 0.749 (0.697-0.801) | 0.597 | 0.196 | 0.693 | 0.609 | 0.455 | 0.830 | 0.521 | 0.180 | 0.758 |
| Clinical external validation cohort (n=335; events=123) | Youden index | 0.304 | 0.749 (0.697-0.801) | 0.597 | 0.196 | 0.663 | 0.530 | 0.724 | 0.627 | 0.612 | 0.180 | 0.758 |
| Clinical external validation cohort (n=335; events=123) | Maximum F1 | 0.304 | 0.749 (0.697-0.801) | 0.597 | 0.196 | 0.663 | 0.530 | 0.724 | 0.627 | 0.612 | 0.180 | 0.758 |

Accuracy, precision, sensitivity, specificity, and F1 score are reported as proportions. The Youden-index and maximum-F1 thresholds were determined from pooled out-of-fold predictions in the development cohort and then fixed for temporal and clinical external validation cohorts. AUROC, area under the receiver operating characteristic curve; AUPRC, area under the precision-recall curve; CI, confidence interval.


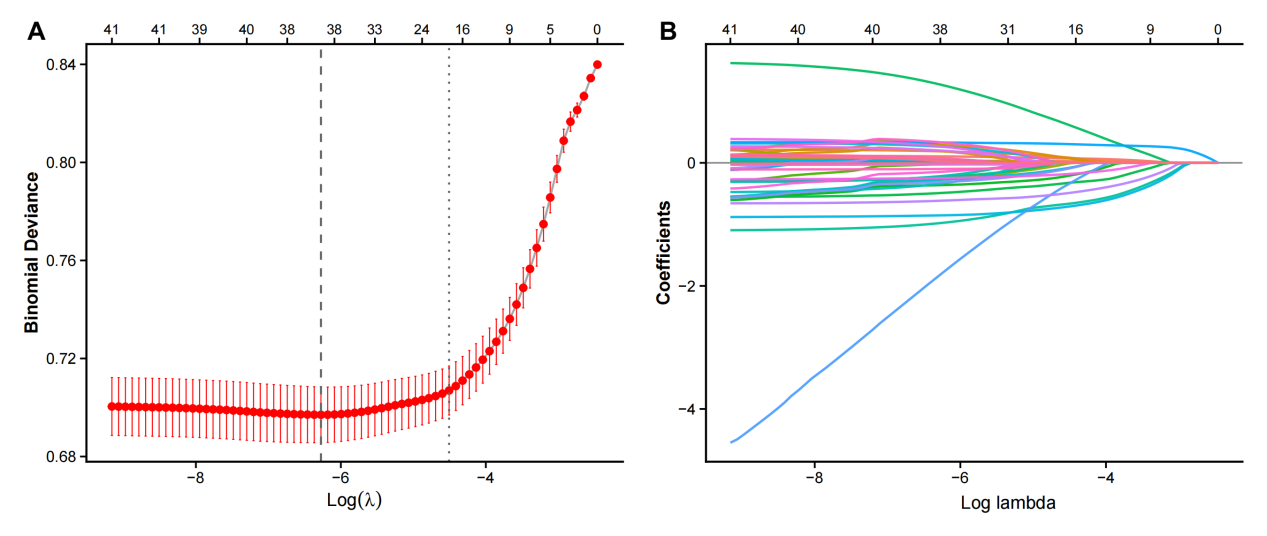


**Supplementary Figure 1.** Feature selection using LASSO regression in the mild insomnia subgroup. (A) Ten-fold cross-validation was used to determine the optimal penalty parameter (λ). The y-axis represents binomial deviance, and the upper x-axis indicates the number of non-zero coefficients. (B) LASSO coefficient profiles of candidate predictors plotted against log(λ). Each colored line represents the coefficient trajectory of one predictor as λ changes.


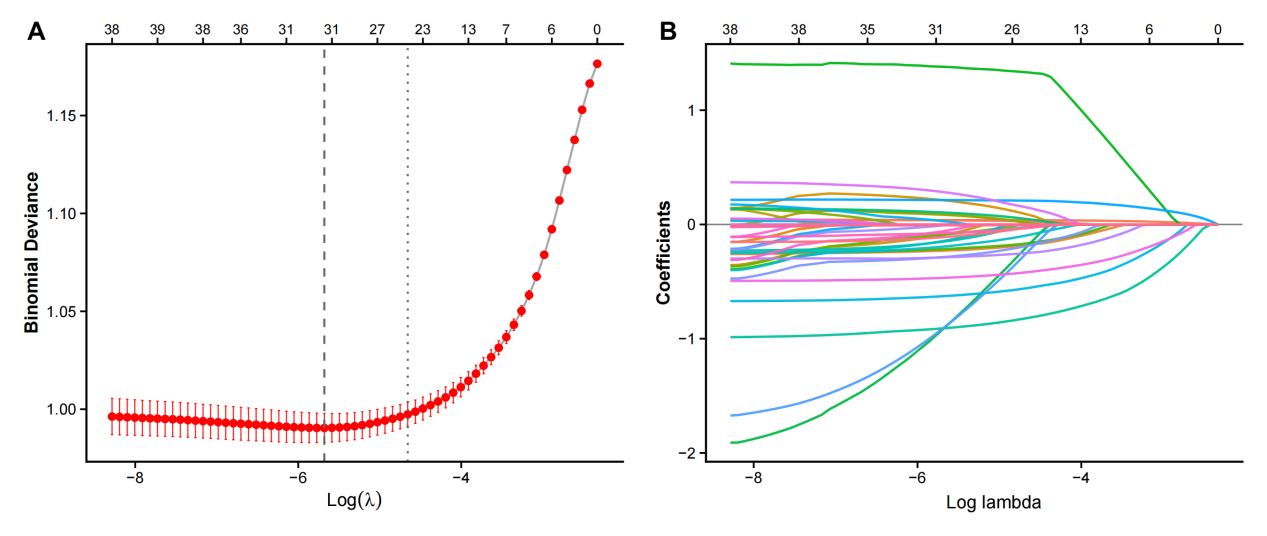


**Supplementary Figure 2.** Feature selection using LASSO regression in the severe insomnia subgroup. (A) Ten-fold cross-validation was used to determine the optimal penalty parameter (λ). The y-axis represents binomial deviance, and the upper x-axis indicates the number of non-zero coefficients. (B) LASSO coefficient profiles of candidate predictors plotted against log(λ). Each colored line represents the coefficient trajectory of one predictor as λ changes.


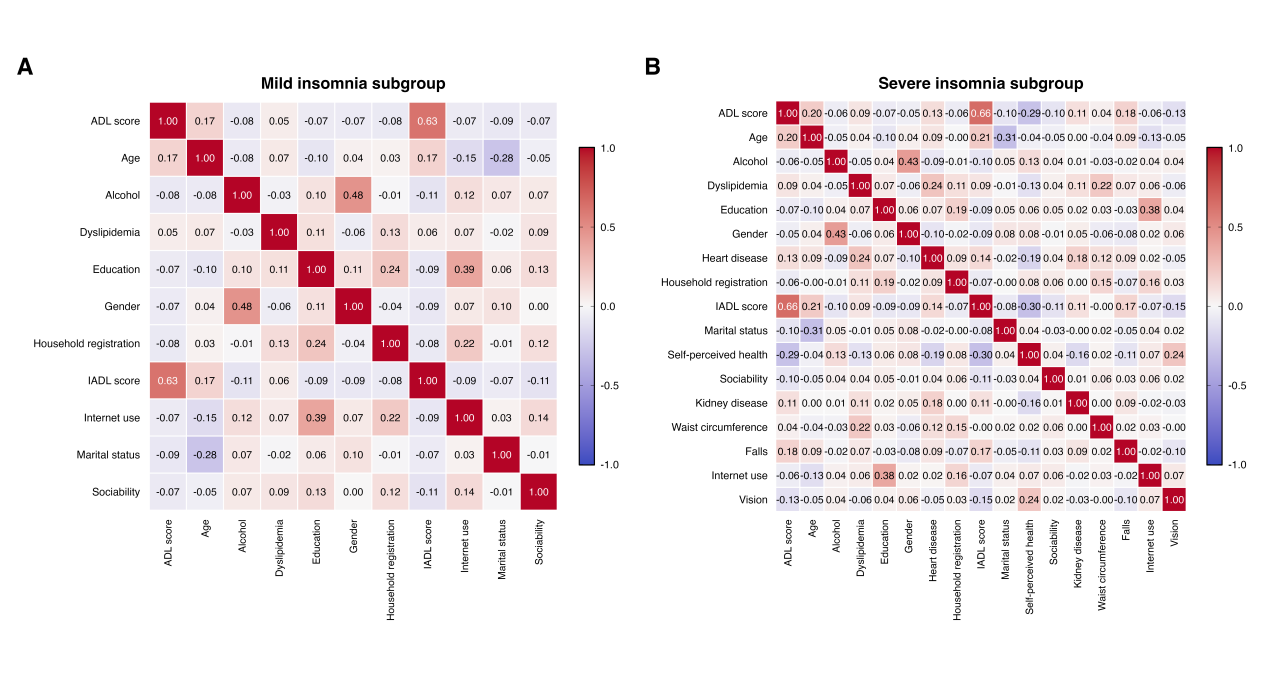


**Supplementary Figure 3.** Pearson correlation matrices of predictors included in the final LightGBM model. (A) Mild insomnia subgroup. (B) Severe insomnia subgroup. The heatmaps display pairwise Pearson correlation coefficients among the predictors. Correlation coefficients are shown within each cell, and color intensity indicates the strength and direction of the association.





**Supplementary Figure 4.** Confusion matrices of the final LightGBM model in the development, temporal validation, and clinical external validation cohorts. (A-C) Mild insomnia subgroup in the development, temporal validation, and clinical external validation cohorts, respectively. (D-F) Severe insomnia subgroup in the development, temporal validation, and clinical external validation cohorts, respectively. The matrices show the numbers of true positives, true negatives, false positives, and false negatives using the default classification threshold of 0.5.





**Supplementary Figure 5.** SHAP dependence plots for the top 9 predictors in the mild insomnia subgroup in the clinical external validation cohort. Each panel shows the relationship between SHAP value (y-axis) and feature value (x-axis) for one predictor. Warmer colors indicate higher feature values, whereas cooler colors indicate lower feature values.





**Supplementary Figure 6.** SHAP dependence plots for the top 9 predictors in the severe insomnia subgroup in the clinical external validation cohort. Each panel shows the relationship between SHAP value (y-axis) and feature value (x-axis) for one predictor. Warmer colors indicate higher feature values, whereas cooler colors indicate lower feature values.


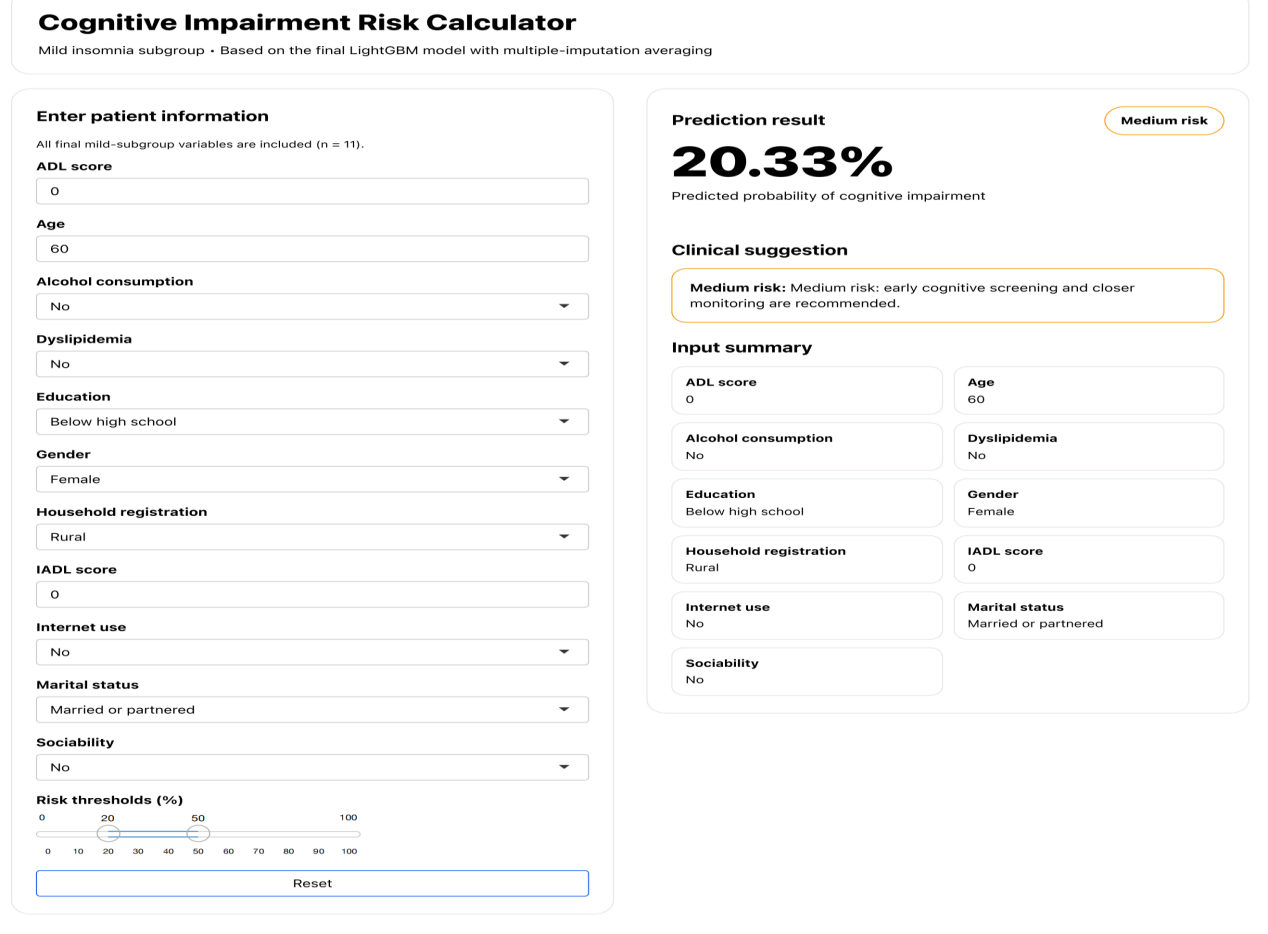


**Supplementary Figure 7.** Web-based calculator for estimating the probability of cognitive impairment in the mild insomnia subgroup based on the final LightGBM model.


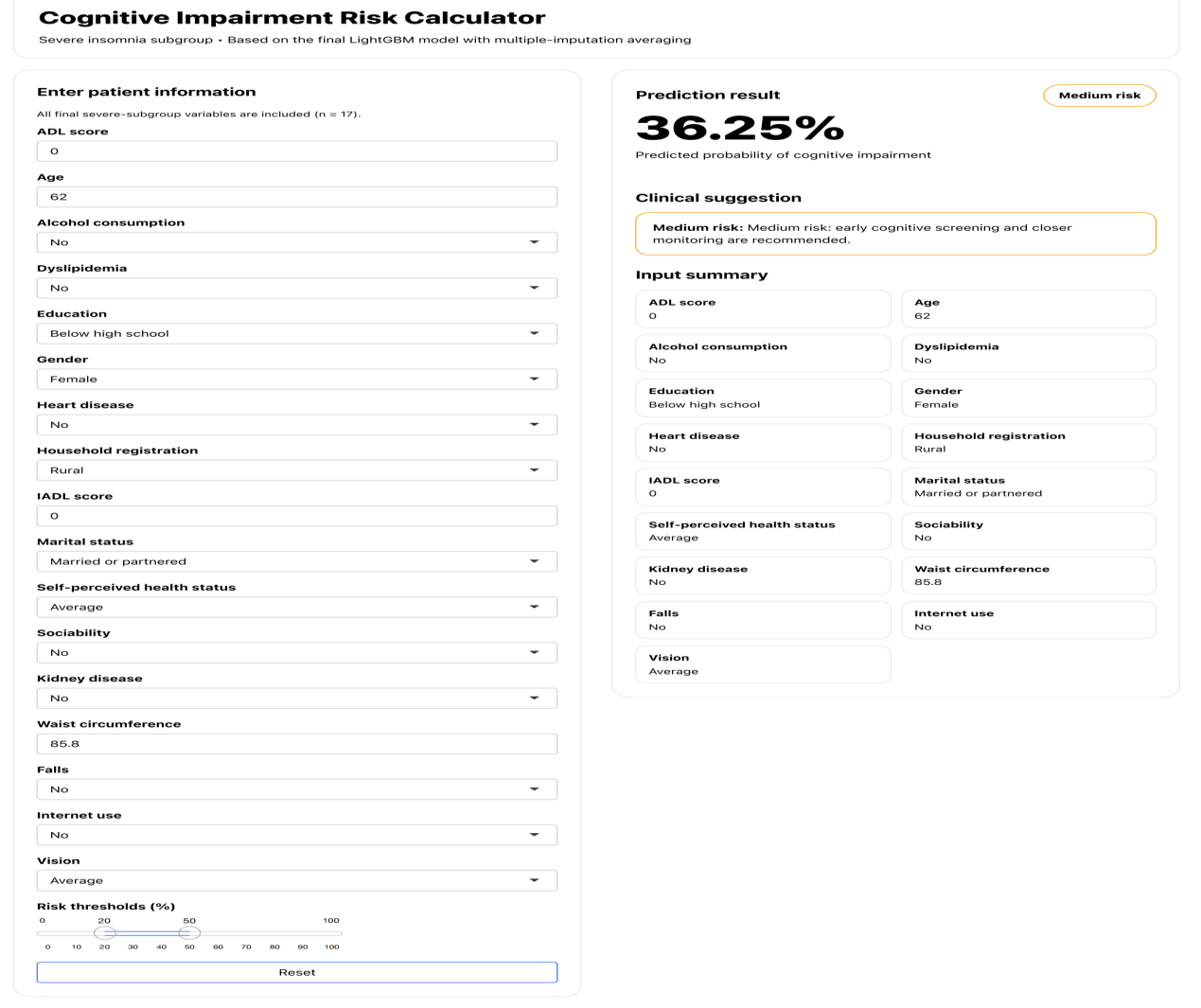


**Supplementary Figure 8.** Web-based calculator for estimating the probability of cognitive impairment in the severe insomnia subgroup based on the final LightGBM model.
